# Dynamic metabolic modeling uncovers systems-level strategies to simultaneously maximize levan yield and substrate efficiency in *Bacillus subtilis* LY7.16

**DOI:** 10.1371/journal.pcbi.1014273

**Published:** 2026-05-18

**Authors:** Muhammad Naufal Hakim, Porntip Chiewchankaset, Saowalak Kalapanulak, Rattiya Waeonukul, Suratsawadee Tiangpook, Treenut Saithong

**Affiliations:** 1 Bioinformatics and Systems Biology Program, School of Bioresources and Technology and School of Information Technology, King Mongkut’s University of Technology Thonburi (Bang Khun Thian), Bangkok, Thailand; 2 Center for Agricultural Systems Biology, Systems Biology and Bioinformatics Research Group, Pilot Plant Development and Training Institute, King Mongkut’s University of Technology Thonburi (Bang Khun Thian), Bangkok, Thailand; 3 Excellent Center of Enzyme Technology and Microbial Utilization, Pilot Plant Development and Training Institute, King Mongkut’s University of Technology Thonburi (Bang Khun Thian), Bangkok, Thailand; Universidade de Vigo, SPAIN

## Abstract

Biopolymers such as levan have attracted growing interest in recent years due to their environmental sustainability and biocompatibility. Nevertheless, industrial-scale production remains constrained by the inherently low yields of bacterial synthesis under mass fermentation conditions. *Bacillus subtilis* LY7.16 was recently identified as a high-performing levan producer, achieving up to 101.9 grams of levan with a substrate use efficiency of 40.7% (w/w). Its scalability is strongly influenced by environmental parameters, particularly the initial sucrose concentration, which regulates both the equilibrium of exopolysaccharide biosynthesis and intracellular metabolic activity. To overcome these limitations, a dynamic metabolic modeling framework (*ly716*-Bs-dMM) was developed to simulate the complex effects of sucrose concentration on substrate conversion to levan. This integrated approach, combining dynamic and constraint-based modeling, incorporates both environmental variables and cellular metabolism to identify strategies for yield enhancement. Model simulations showed strong agreement with experimental data, demonstrating that elevated sucrose concentrations promote levan production while driving a metabolic shift from biomass synthesis toward levansucrase expression, mediated by *sacB* upregulation. Furthermore, the simulations demonstrate transition in extracellular levansucrase activity—from hydrolysis dominance under low sucrose conditions to transfructosylation dominance under high sucrose abundance. Collectively, *ly716*-Bs-dMM underscores a systems metabolic engineering strategy that integrates microbial physiology with operating conditions to simultaneously maximize levan yield and substrate use efficiency. Scenario-based analysis of a predictive hypothesis further suggests that integrating amino acid supplementation with fed-batch operation, alongside energy pathway engineering, can overcome the current tradeoff between levansucrase activity and cell biomass formation, thereby advancing levan production potential.

## Author summary

Biopolymers are increasingly attractive for applications in medicine and the food industry due to their non-toxic and biocompatible properties. Levan is one of holding significant promises, yet its typically low synthesis yield hinders an advancement to industrial applications. *Bacillus subtilis* LY7.16 has been demonstrated as an efficient strain for levan production, offering superior yield and substrate utilization. However, the strong dependence of LY7.16-derived levan on fermentation conditions, particularly sucrose availability, limits its industrial potential unless system-level optimization is applied. To address this, we developed an integrated framework, *ly716*-Bs-dMM, which couples dynamic and constraint-based modeling to simultaneously capture substrate conversion dynamics and cellular metabolism. Simulations revealed that elevated sucrose concentrations enhance levan production by shifting metabolism from biomass synthesis toward levansucrase expression, driven by *sacB* upregulation. Moreover, extracellular levansucrase activity was shown to transition from hydrolysis dominance under low sucrose to transfructosylation dominance under high sucrose abundance. Further analysis of *ly716-*Bs-dMM offers the systems metabolic engineering scenario for optimizing levan by amino acid supplementation and gene downregulation. Overall, our framework successfully simulates scenario-based circumstances of levan synthesis in *B. subtilis* LY7.16 that effectively facilitate maximum levan synthesis, along with substrate efficiency to real-world implementation in industrial sector.

## Introduction

Biopolymers have attracted attention nowadays because of their superior performance over fossil-based polymers, particularly in biocompatibility and environmental friendliness [1,2]. Levan biopolymer has a wide range of applications ranging across biomedical to cosmetic industries [3,4]. Levan is a homopolysaccharide of fructose produced by plants and microbes [2,5,6]. Microbial levan is typically produced within days, which is significantly faster than plant-based levan, which requires at least a few months [2,6,7]. In general, microbial levan is synthesized extracellularly via the secreted levansucrase enzymatic protein. The enzyme is expressed from the *sacB* gene [8,9] and its activity is highly influenced by sucrose availability during fermentation process [10,11]. Although levan can be rapidly produced by microorganisms, the large-scale production is often constrained by low yields and high costs associated with inefficient substrate utilization [4,12]. These challenges stem from sub-optimal operations, frequently caused by the isolated optimization of microbial cell characteristics and operating parameters of production process. To scale up microbial levan production to an industrial level, a more integrated approach is required—one that combines the selection of highly efficient microbial strains with the precise tuning of operating conditions.

*Zymomonas* and *Bacillus* are well-known bacterial genera associated with various high-performance microbial levan producers [13–16]*.* Their ability to synthesize levan, coupled with their classification as generally recognized as safe (GRAS) organisms, makes them ideal candidates for applications catering to human needs. *Zymomonas* is capable of producing levan up to 0.32 g_levan_ · g_sucrose_^-1^ (~ 0.13-0.32 g_levan_ · g_sucrose_^-1^), while *Bacillus* can achieve production levels as high as 0.4 g_levan_ · g_sucrose_^-1^ (~ 0.06-0.40 g_levan_ · g_sucrose_^-1^) [14–17]. *B. subtilis* LY7.16 is one of the outstanding levan-producing strain previously isolated from Thai traditional fermented soybean and maintained in the culture collection of the Excellent Center of Enzyme Technology and Microbial Utilization, King Mongkut’s University of Technology Thonburi (KMUTT) [18]. The strain was deposited at the Thailand Bioresource Research Center (TBRC), Thailand, under accession number TBRC 11053. *B. subtilis* LY7.16 exhibits strong levan-producing capability, achieving a maximum yield of 0.43 g_levan_ · g_sucrose_^-1^ at an initial sucrose concentration of 250 g L ⁻ ¹. Nevertheless, the potential of microbial strains is always evaluated particularly under ideal laboratory conditions which weakly represent the complex and variable environments encountered during large-scale fermentation. Such discrepancies impede the attainment of optimal yields and ultimately constrain the strains’ effectiveness as industrial-grade levan producers.

On the other hand, key fermentation parameters are typically optimized to identify operating conditions that maximize levan productivity [19]. Studies have shown the major factors include initial sucrose concentration and temperature [13,20–22]. Among these environmental factors, initial sucrose concentration is the most influential in levan synthesis, as it serves as the primary substrate and activates levansucrase expression [2,3]. Literature indicates that sucrose availability in levan production not only affects substrate supply but also plays a crucial role in the sucrose-dependent activity of levansucrase [5,13,22–24]. At a given enzyme concentration, higher sucrose levels correlate with reduced hydrolysis reactions, ultimately enhancing levan yield [25,26]. Initial sucrose concentration is, thus, considered as a primary optimizing-factor for levan yield enhancement. Temperature is also a critical factor in regulating the balance between sucrose hydrolysis and sucrose transfructosylation activities [5,13,27], where levan synthesis occurs exclusively via transfructosylation during production [22].

Maximizing levan production to achieve its full potential requires a synergistic strategy that integrates microbial cell metabolism with environmental factors. Wu et al. [13] optimized levan production by *B. subtilis* Natto through adjusting environmental factors, including initial sucrose concentration, temperature and pH. Their study demonstrated that the yield product per substrate (*Y*_*P/S*_) of *B. subtilis* Natto increased to 0.35 g_levan_ · g_sucrose_^-1^ under the optimized conditions. More recently, Mu et al. [28] found that the increase of levan production by initial sucrose concentration is restricted to the potent of microbial cell metabolism to utilize sucrose substrate in equilibria to extracellular conversion of sucrose by microbial levansucrase. By contrast, Gu et al. [16] explored enhancing levan yield in *B. amyloliquefaciens* through metabolic engineering and achieved a 2.14-fold increase from the type of strain, but under the ideal controlled environmental setting. These findings underline the need of a combined strategy addressing both microbial metabolism and environmental factors, as they are essential for enhancing levansucrase activity and maximizing levan synthesis [4,29]. However, the current experimentation techniques are hard to implement and weakly capture the dynamic interaction of microbial cell performance and environmental influences, especially under time-evolving fermentation conditions.

Computational modeling offers a powerful approach for simultaneously investigating microbial metabolic potential and environmental conditions within a unified system optimization framework. In particular, dynamic metabolic modeling enables the prediction of metabolic shifts in response to changing environmental factors throughout the fermentation process [30–32]. This approach integrates ordinary differential equation (ODE)-based models with genome-scale metabolic models (GEMs), providing a comprehensive platform for simulating and optimizing microbial behavior under dynamic conditions. This method employs a set of ODEs to describe dynamic changes in extracellular metabolites over time. The auxiliary parameters derived from ODEs serve as constraints in GEMs, which estimate intracellular flux distribution throughout fermentation. This integrated framework effectively links environmental factors with microbial metabolism over time, thereby enhancing our integral understanding of their interdependent influences on production yield. Nevertheless, there was no hybrid ODE-GEMs model applied to levan biosynthesis to date. The closest modeling approach that already being applied was in *Zymomonas mobilis* B-14023, where the RSM-modeling based approach was used to optimize fermentation time, initial substrate concentration, and pH [19]. Studies involving high-performance *Bacillus subtilis* are even less. Moreover, most existing work focuses either solely on identifying optimal operational parameters while neglecting cellular metabolic constraints, or on proposing potential metabolic engineering targets without considering the environmental conditions [20,33].

In this study, we employed a dynamic metabolic modeling approach to investigate a strategy for maximizing yield while minimizing substrate use in levan production by *B. subtilis* LY7.16. A dynamic metabolic model of *B. subtilis* LY7.16, denoted as *ly716*-Bs-dMM, was developed to simulate microbial growth and changes of metabolite profiles over time in varying initial sucrose concentrations, ranging from 100 g · L^-1^ (low concentration), 200 g · L^-1^ (low-to-high transitional condition), to 250 g · L^-1^ (high concentration). The simulation results demonstrate the hypothetical shift in extracellular levansucrase activity, from hydrolysis dominance under low sucrose conditions to transfructosylation dominance under high sucrose abundance. High initial sucrose concentrations were also shown to influence intracellular metabolic fluxed in *B. subtilis* LY7.16, redirecting elevated metabolic activity from biomass synthesis toward levansucrase production, driven by enhanced *sacB* expression. Finally, the modeled results offered the strategy derived by a predictive hypothesis for the levan enhancement scenario that yielded from the analysis of the integrated cell and operating environment factors.

## Results

### Levan production by *B. subtilis* LY7.16 under different initial sucrose concentrations

**Fig 1:**
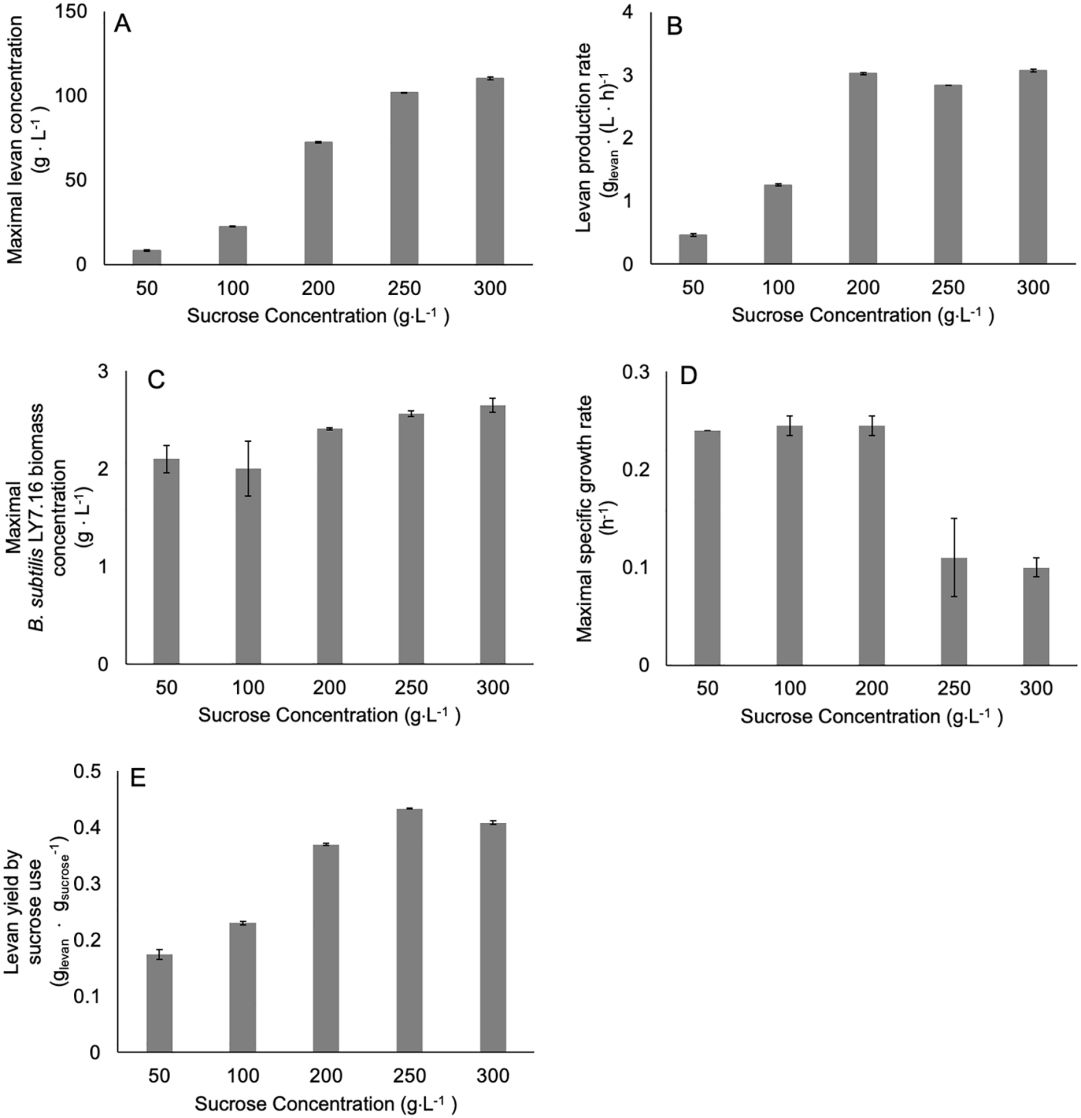
Levan production by *B. subtilis* LY7.16 under varying initial sucrose concentrations, (A) maximal levan concentration, (B) levan production rate, (C) maximal *B. subtilis* LY7.16 biomass concentration, (D) maximal specific growth rate, and (E) levan yield by sucrose use.

Levan production is primarily influenced by substrate concentration and the metabolic potential of the producing microorganism. Drawing data from levan synthesis under varying sucrose concentrations (Fig A in S1 Text), we examined the interplay between these factors to identify strategies for surpassing current production limits. The primary analysis of levan production by *B. subtilis* LY7.16 showed that the maximal levan production positively correlated with the increase of initial sucrose concentration. The association showed steeply increased levan production up to 200 g · L^-1^ of initial sucrose concentration, then steadily when concentration greater than 250 g · L^-1^ (Fig 1A and 1B). The changes in rate of levan production upon the given sucrose concentration are corresponding to the decline of *B. subtilis* LY7.16 growth rate (Fig 1B and 1D). The results showed that cell biomass production rate steadily high at sucrose concentrations 50 g · L^-1^ to 200 g · L^-1^ and started decreasing at 250 g · L^-1^ (Fig 1D). At the high sucrose concentrations (250 g · L^-1^ to 300 g · L^-1^), *B. subtilis* LY7.16 exhibited slow biomass production, which were presumed to result in lower levansucrase levels. However, under this condition, the highest levan yield by substrate use was apparently observed (Fig 1E and Table 1). The contrasting effects of sucrose concentration on maximal levan production and cell biomass formation create a complex challenge in achieving the optimal balance of these influential factors for levan production. This phenomenon is hypothesized to result from differences in the equilibrium states of sucrose utilization for *B. subtilis* LY7.16 growth and its availability for levan synthesis via extracellular levansucrase at varying sucrose concentrations.

**Table 1:** Analysis of levan production by *B. subtilis* LY7.16 under varying initial sucrose concentrations.

Sucrose concentration (g ⋅ L^-1^)	Maximal levan concentration (g ⋅ L^-1^)	Levan production rate (g·(L·h)^-1^)	Time to reach maximal levan concentration (h)	Maximal *B. subtilis* LY7.16 biomass concentration (g ⋅ L^-1^)	Maximal specific growth rate (h^-1^)	Levan yield by sucrose use (g_levan_ ⋅ g_sucrose_^-1^)
50	8.30 ± 0.42	0.461 ± 0.02	18.0	2.10 ± 0.14	0.240 ± 0.00	0.175 ± 0.01
100	22.6 ± 0.28	1.256 ± 0.02	18.0	2.00 ± 0.28	0.245 ± 0.01	0.230 ± 0.00
200	72.5 ± 0.42	3.021 ± 0.02	24.0	2.41 ± 0.01	0.245 ± 0.01	0.370 ± 0.00
250	101.9 ± 0.14	2.831 ± 0.00	36.0	2.56 ± 0.03	0.11 ± 0.04	0.433 ± 0.00
300	110.4 ± 0.85	3.067 ± 0.02	36.0	2.65 ± 0.07	0.10 ± 0.01	0.408 ± 0.00

Metabolite profiling during levan production (Fig A in S1 Text) suggested that the equilibrium shift is likely to occur when initial sucrose concentrations were greater than 200 g · L^-1^. At low range of sucrose concentrations (50 g · L^-1^ and 100 g · L^-1^), sucrose was rapidly converted and showed a sharp increase of cell biomass production and levan within a short time. This rapid conversion occurred only momentarily due to lacking sucrose to sustain biomass and levan production (Fig A (A, B) in S1 Text). Following sucrose depletion, an increase in fructose levels was observed, accompanied by a decline in cell biomass and levan. This phenomenon may be attributed either to insufficient levansucrase production by *B. subtilis* LY7.16 or to levansucrase-mediated levan degradation as a compensatory response, wherein the enzyme utilizes accumulated levan as an alternative fructose source under sucrose-limited conditions. In this regard, sucrose availability plays a crucial role in levan production, as it not only promotes levan synthesis but also prevents its degradation. At high range of sucrose concentrations (250 g·L^-1^ and 300 g·L^-1^), sucrose gradually decreased during the initial phase before undergoing a steep decline, while fructose showed significantly decreased then slowly rebounded after sucrose depletion. These changes in substrate profiles were associated with a slower rate of cell biomass and levan production and decay compared to the observation at lower range of sucrose concentrations (Fig A (C-E) in S1 Text). The high sucrose availability likely hindered *B. subtilis* LY7.16 growth during the early period, as observed deceleration in growth rate compared to lower concentrations, but the initial futile level of sucrose appeared to mitigate the sharp degradation of levan after sucrose depleted. The results suggested distinct mechanisms of substrate conversion and possibly differences in intracellular metabolic activity between low and high initial sucrose concentrations, ultimately influencing total yield and substrate utilization efficiency. These findings highlight the strong dependence of levan production on the balance between sucrose utilization for cell growth and its conversion into levan, which was greatly influenced by the initial sucrose availability.

### Dynamic metabolic modeling of levan production by *B. subtilis* LY7.16 under low and high sucrose culture conditions

**Fig 2:**
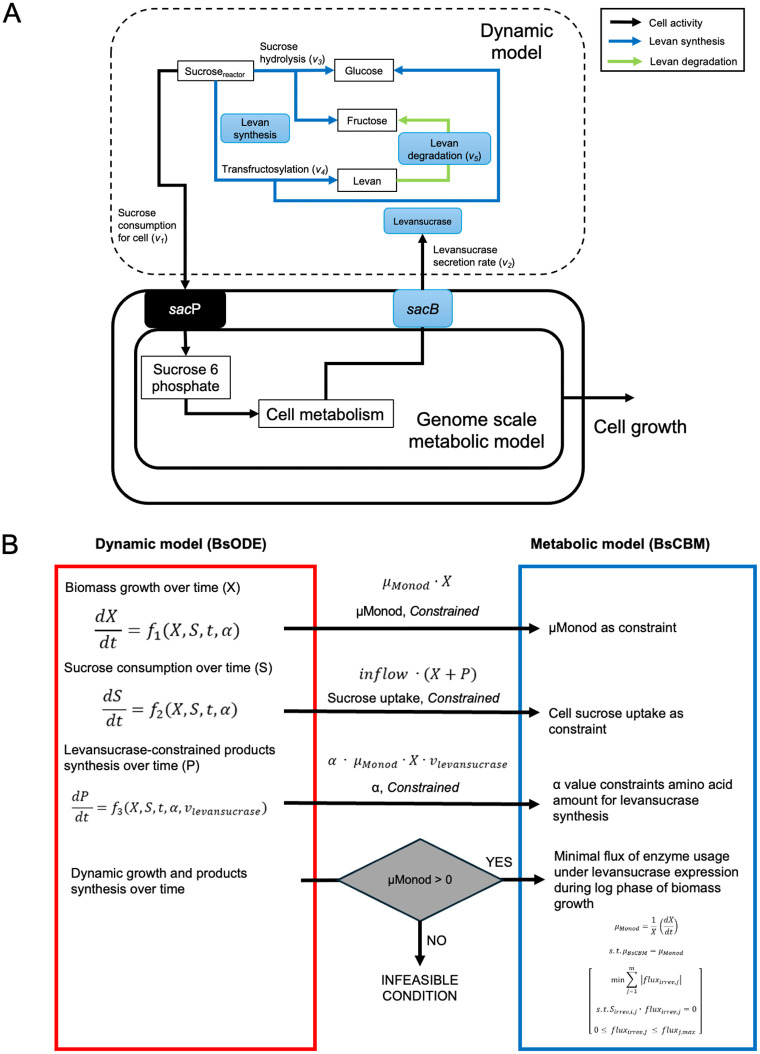
Scheme of a dynamic metabolic modeling framework of levan production by *B. subtilis* LY7.16 (*ly716*-Bs-dMM). (A) Hypothetical cascade illustrates a process-based model of levan production by *B. subtilis* LY7.16. All rates of metabolite conversion (*v*) are presented in mmol·(L·h)^-1^. Intracellular metabolic fluxes and cell growth rate are presented in mmol·(gDW·h)^-1^ and h^-1^, respectively. (B) The integrated dynamic metabolic modeling framework demonstrates the coupling of the dynamic (BsODE) and genome-scale metabolic (BsCBM) models in representing the association of extracellular substrate conversion and microbial metabolic-dependent levansucrase production in levan synthesis.

To maximize levan yield and ensure efficient substrate utilization by *B. subtilis* LY7.16, a process-based model of levan production was developed, as illustrated in Fig 2. The hypothetical cascade of levan production by *B. subtilis* LY7.16 described the conversion of sucrose as a substrate for microbial levansucrase productions and extracellular levan synthesis via the secreted levansucrase enzyme (Fig 2A). These processes are proposed as key determinants of final levan yield and substrate utilization efficiency. The presence and level of sucrose concentrations triggered the expression of *sac*B gene that encoded levansucrase enzyme, which was secreted outside the cell. Levansucrase converts sucrose via two biochemical reactions: hydrolysis – breaking down sucrose into fructose and glucose, and transfructosylation – converting sucrose to levan and glucose. The apparent antagonistic relationship of levan synthesis by levansucrase and the sucrose-dependent enzyme production in *B. subtilis* LY7.16 introduces complexity in optimizing the levan production process to achieve both efficiency and effective substrate utilization.

The integrated dynamic metabolic modeling framework, denoted as *ly716*-Bs-dMM (Fig 2B), combines dynamic (BsODE) and genome-scale metabolic models (BsCBM) to simultaneously analyze environmental and cellular metabolism under specific conditions. The BsODE model integrates extracellular reactions (Fig 2A) to simulate microbial biomass growth, sucrose consumption, and the synthesis of glucose, fructose, and levan under constrained parameters. This framework enables simulation of dynamic profiling of metabolite concentrations over time and facilitates the prediction of intracellular metabolic responses to varying initial sucrose concentrations.

**Fig 3:**
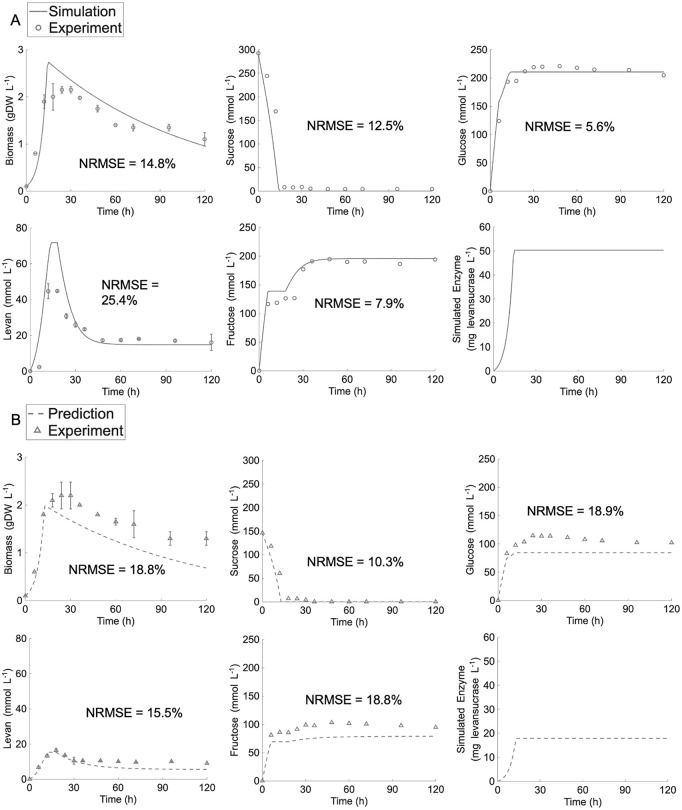
Simulation of *ly716*-Bs-dMM under low range of initial sucrose concentrations. (A) Model simulation at initial sucrose concentration of 100 g·L^-1^. (B) Model prediction for validation the simulatability of *ly716*-Bs-dMM under initial sucrose concentration of 50 g·L^-1^ condition.

The *ly716*-Bs-dMM was first employed to simulate sucrose conversion into levan synthesis at an initial sucrose concentration of 100 g·L^-1^. The simulation results in Fig 3A demonstrated that the model effectively captured sucrose consumption in relation to microbial cell biomass and levan production, though it slightly over-estimated the maximal levan production in this framework. The model also successfully simulated changes in all relevant biochemical concentrations during levan production. The simulation revealed that sucrose was rapidly hydrolyzed into glucose and fructose, and was also efficiently utilized by *B. subtilis* LY7.16. Its rapid consumption led to the swift accumulation of fructose substrate and levansucrase enzyme, facilitating levan synthesis. As sucrose levels declined, fructose remained steady while glucose continued to increase, indicating heightened transfructosylation activity in converting fructose to levan after six hours. Unlike hydrolysis, transfructosylation produces both levan and glucose and is significantly influenced by sucrose and levansucrase availability. Upon sucrose depletion, cell growth declined, and cell-secreted levansucrase enzyme was predicted to level off. During this phase, levan production appeared to decrease, in contrast to a rise in fructose levels. This shift suggested a reversal in the levansucrase-mediated reaction, wherein the degradation of existing levan into fructose helped sustain equilibrium between cell growth and levan synthesis. The ability of levansucrase to degrade levan into its fructose monomer has been well established [10,34]. Recent studies indicate that levan degradation progresses only up to a certain level and does not achieve complete conversion, likely due to the chemical structure of levan [34]. As a result, levan degradation and the subsequent synthesis of fructose reached a steady state once this condition was met.

The predictive performance of *ly716*-Bs-dMM at low range of substrate concentration was evaluated using an independent experiment of levan production that was initialized at sucrose concentration of 50 g·L^-1^ (Fig 3B). Similar phenomena were observed, with a less rapid sucrose consumption under this condition. During biomass growth and levansucrase secretion, hydrolysis exhibited a sharp increase at the sixth hour, after which transfructosylation became the dominant reaction, rapidly producing levan and glucose. Additionally, once levan reached its maximum concentration, it underwent degradation, accompanied by an increase in fructose levels. The *ly716*-Bs-dMM model successfully captured these key phenomena, ensuring accurate representation at low sucrose concentrations. It is worth noting that while overall metabolite conversion was well simulated, the model appeared to underestimate microbial cell growth and subsequent levan-associated products, including glucose and fructose. This difference may suggest that levansucrase activity in the model may lower than in reality under these conditions. The average normalized root mean squared error (NRMSE), which quantifies the deviation of model simulations from experimental values, was comparable between the 50 g·L^-1^ (16.46%) and 100 g·L^-1^ (13.24%) conditions. These results demonstrate that the *ly716*-Bs-dMM model with the optimal parameter values (*α*, = 18.19 mg_levansucrase_·gDW_biomass_^-1^ and *v*_*max,trans,levansucrase*_ = 6.42 mmol·mg_levansucrase_^-1^ h^-1^*, µ*_*max*_ = 0.2454 h^-1^, Table A in S2 Text) can robustly simulate sucrose conversion into levan across a low range of initial sucrose concentrations.

**Fig 4:**
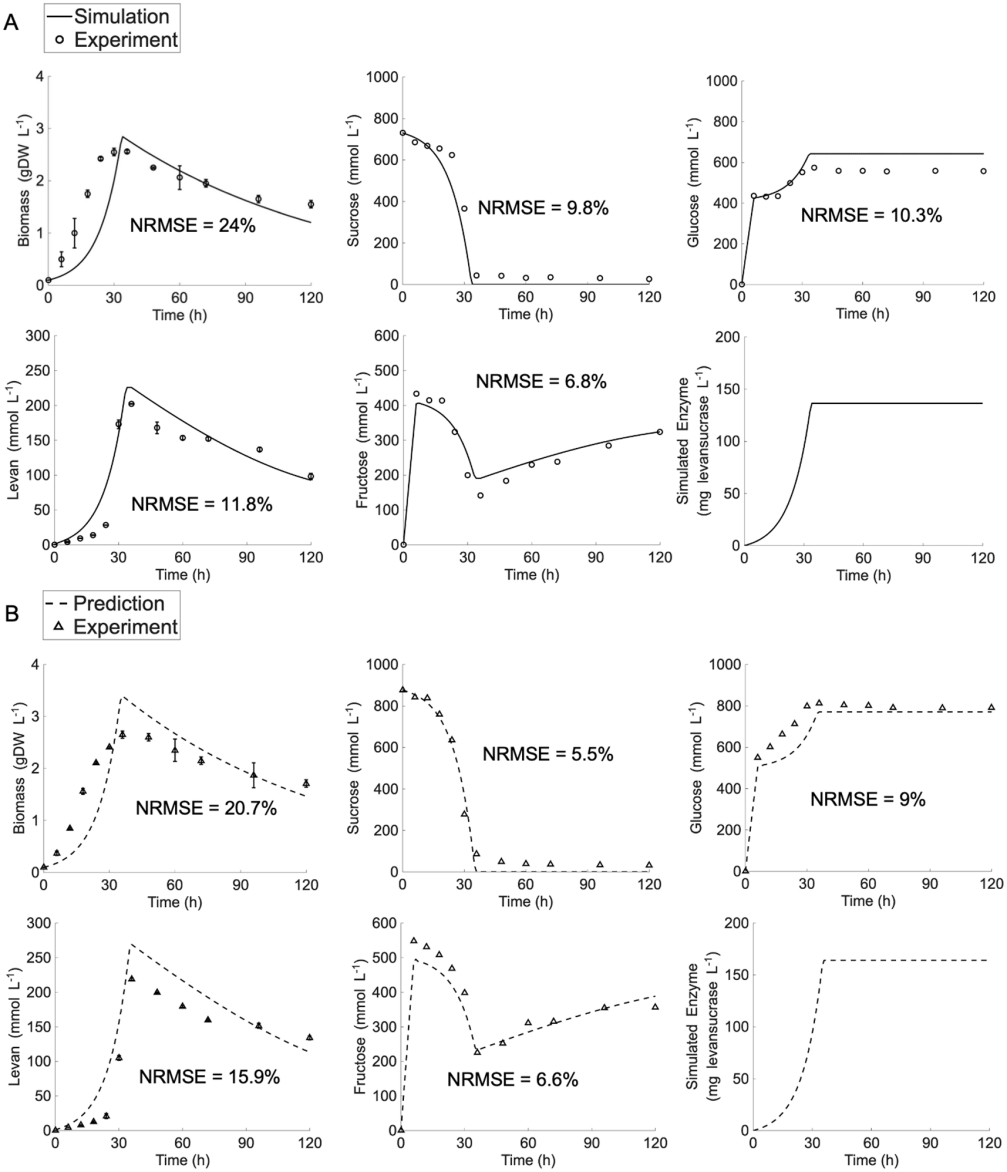
Simulation of *ly716*-Bs-dMM under high range of initial sucrose concentrations. (A) Model simulation at initial sucrose concentration of 250 g·L^-1^. (B) Model prediction for validation the simulatability of *ly716*-Bs-dMM under initial sucrose concentration of 300 g·L^-1^ condition.

The *ly716*-Bs-dMM was further applied to simulate levan production under high initial sucrose concentration, primarily at 250 g·L^-1^. The simulation presented in S1 Text (Fig B) deviated from the experimentally measured metabolite profiles, indicting limitations of the current *ly716*-Bs-dMM framework in accurately representing sucrose-to-levan conversion dynamics under high initial sucrose concentrations. The deviations were particularly evident in the profiles of levan, sucrose, and especially fructose, for which the simulated patterns diverged noticeably from the experimental measurements. The model was able to mimic the initial sharp increase of fructose levels (0–6 h), but failed to reproduce the subsequent rapid decline and the later rebound in accumulation following sucrose depletion (6–36 h). The distinct fructose conversion dynamics under low and high initial sucrose concentrations indicate divergent equilibrium states governing sucrose utilization toward levan synthesis and microbial-mediated levansucrase production. This behavior may be attributed to altered transfructosylation activity and changes in microbial metabolism under the sucrose abundance gradient. To test the hypothesis, the *ly716*-Bs-dMM model was reparametrized to reflect sucrose-dependent transfructosylation activity in levan biosynthesis (*α*, = 45 mg_levansucrase_·gDW_biomass_^-1^ and *v*_*max,trans,levansucrase*_ = 6.42 mmol·mg_levansucrase_^-1^ h^-1^, Table A in S2 Text), along with microbial metabolic adaptation in response to elevated sucrose availability (*µ*_*max*_ = 0.1 h^-1^, Table A in S2 Text). Fig 4A showed that the re-optimized *ly716*-Bs-dMM model successfully captured key dynamic behaviors under high initial sucrose concentration, including sucrose consumption, biomass production, levan production, and changes in fructose and glucose concentrations. The NRMSE of model simulation against measured profiles ranged from 6.8% to 24%, with average NRMSE in high concentration is 12.54% for 250 g·L ⁻ ¹ and 11.54% for 300 g·L ⁻ ¹. Moreover, the reliability of the *ly716*-Bs-dMM model was further reinforced by its validated simulation of levan production at an initial sucrose concentration of 300 g·L ⁻ ¹ (Fig 4B). These validation results demonstrated that the re-optimized model can robustly simulate levan production dynamics, covering high range of initial sucrose concentrations with NRMSE values from 5.5% to 20.7%.

Overall, the *ly716*-Bs-dMM model effectively simulated sucrose-to-levan production across a broad range of initial sucrose concentrations. The optimal parameter sets tailored to initial sucrose concentrations revealed a shift in the levansucrase-mediated biochemical conversion pathway, underpinning the distinct conversion dynamics observed in levan synthesis initiated under low versus high sucrose conditions. In addition, the optimal parameters which were obtained from fitting shown the consistency with previously reported enzyme kinetics (S2 Text), indicating the levansucrase mechanism is also consistent with prior study [22]. These results underscore the critical importance of fine-tuning initial sucrose levels to maximize yield within a practical timeframe and emphasize the necessity of balancing the unique mechanistic advantages offered by both low and high sucrose availability.

### Simulation of B. subtilis LY7.16-based levan production under low-to-high transitional sucrose concentrations

The optimal initial substrate concentration has been shown to determine maximal yield, substrate use efficiency, and production rate. As shown in Table 1, a shift in sucrose conversion efficiency and levan production was observed between the low (50–100 g·L ⁻ ¹) and high (250–300 g·L ⁻ ¹) initial sucrose concentration ranges. This shift resulted in a 5- to 12-fold increases in maximal levan production, albeit with approximately twice the production time due to limited levansucrase abundance from the ~ 2.5-time reduction in growth rate of *B. subtilis* LY7.16 under the high sucrose concentration. The appearing trade-off phenomena over the range of initial sucrose concentrations pinpointed an optimal concentration near the transition between low and high levels, potentially around 200 g·L ⁻ ¹.

**Fig 5:**
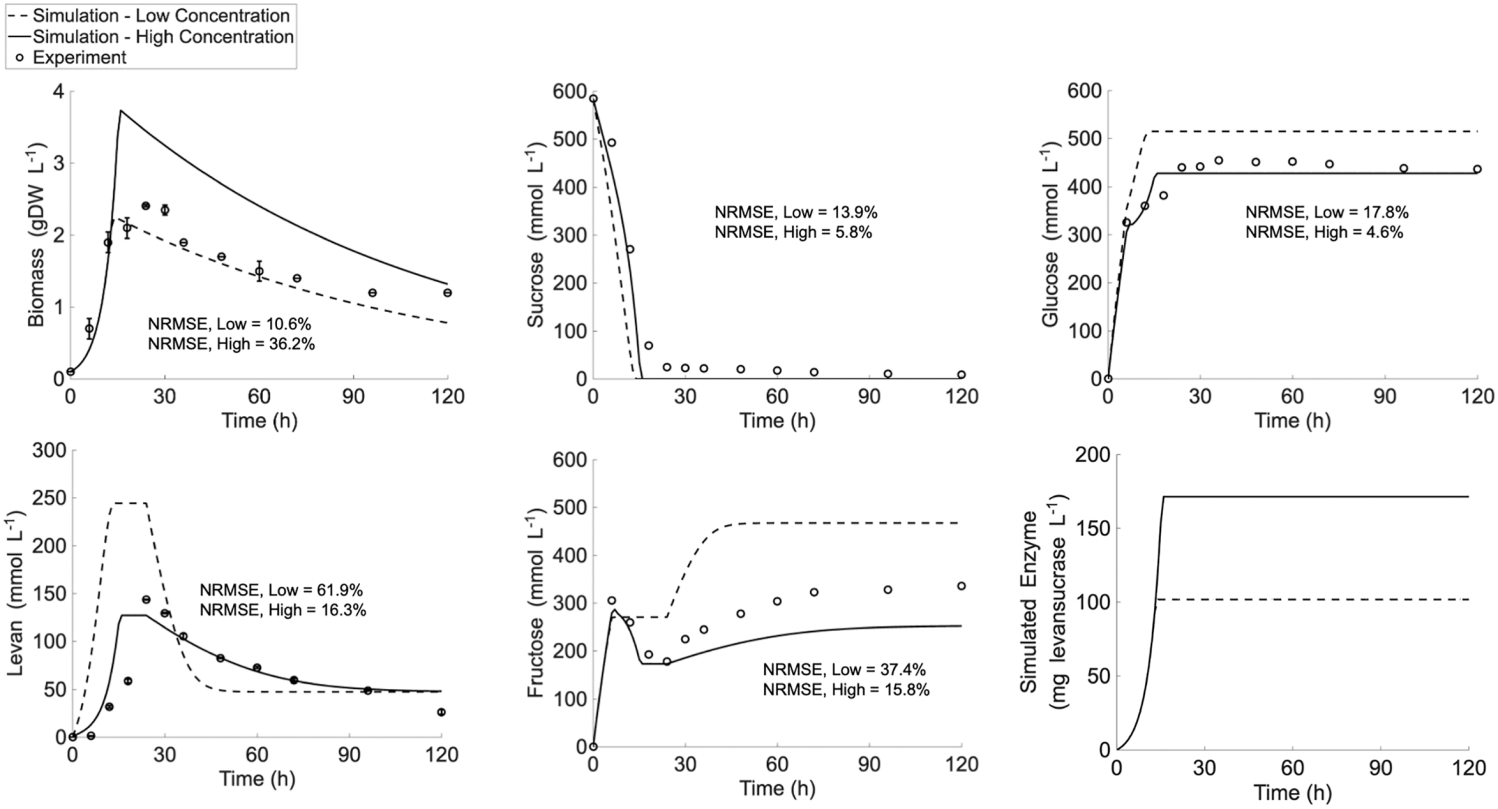
Simulation of *ly716*-Bs-dMM at initial sucrose concentration of 200 g·L^-1^ based upon the condition-specific parameters, dash line – low initial sucrose concentration and solid line – high initial sucrose concentration. The results demonstrate the marginal range of low to high initial sucrose substrate concentration in effect to sucrose-to-levan conversion equilibrium.

At an initial sucrose concentration of 200 g·L ⁻ ¹, the time-course profiles of all biochemical conversions exhibited intermediate behavior relative to those observed across the substrate concentration gradient (Fig A in S1 Text), consistent with its corresponding yield performance (Table 1). To assess whether the conversion process at 200 g·L ⁻ ¹ sucrose reflects a mechanistic blend of low- and high-range dynamics, the *ly716*-Bs-dMM model was employed to simulate levan production using parameter sets specific to each condition. The simulated result in Fig 5 showed that the measured metabolite conversion at 200 g·L ⁻ ¹ (Fig 5 (dot)) was only partially mimicked by either low-range (Fig 5 (dash line)) or high-range (Fig 5 (solid line)) modeling scenarios, and exhibited slightly closer resemblance to the high-range condition. Re-parameterization of the *ly716-*Bs-dMM model to improve its representation of sucrose-to-levan conversion at an initial sucrose concentration of 200 g·L ⁻ ¹ yielded optimized parameters indicative of a transitional state between low and high sucrose regimes (Table A in S2 Text). This is reflected in a combined profile of key conversion parameters: a biomass yield on substrate (*Y*_*X/S*_)) of approximately 0.015 gDW_biomass_·mmol sucrose ⁻ ¹, positioned between optimal values for low and high concentrations; an *α* (45 mg_levansucrase_·gDW_biomass_ ⁻ ¹) value resembling that of the high sucrose condition; and a *μ*_*Monod*_ (0.2454 h^-1^) value closely aligned with the low concentration regime. These parameter values suggest that at this transitional concentration, *B. subtilis* LY7.16 may begin shifting its metabolic priorities from biomass production toward levansucrase synthesis to supply the higher abundance of sucrose. While entering to the high range of initial sucrose conditions, demand for levansucrase is greater upon the initial sucrose availability. It led to a rebalancing metabolic equilibrium of *B. subtilis* LY7.16 to reduce biomass growth for levansucrase production. This hypothetical scenario suggests that the microbial metabolic potential and optimization upon the initial substrate concentration is a critical factor for leaping forward the overall performance of levan production.

### Prediction of metabolic alteration in *B. subtilis* LY7.16 upon initial sucrose concentration

Simulations of the *ly716*-Bs-dMM model demonstrated that initial sucrose concentrations influenced levan synthesis not only through substrate availability, but potentially also via modulating microbial levansucrase production. Optimized values of key parameters—*μ*_*Monod*_, *Y*_*X/S*_, and *α*—across varying initial sucrose concentrations reveal metabolic adaptations in *B. subtilis* LY7.16 that promote levansucrase synthesis under elevated sucrose availability. To investigate potential trade-offs between microbial cell biomass accumulation and levansucrase production across sucrose gradients, carbon flux distributions in *B. subtilis* LY7.16 were analyzed to elucidate metabolic responses to changing substrate levels.

**Fig 6:**
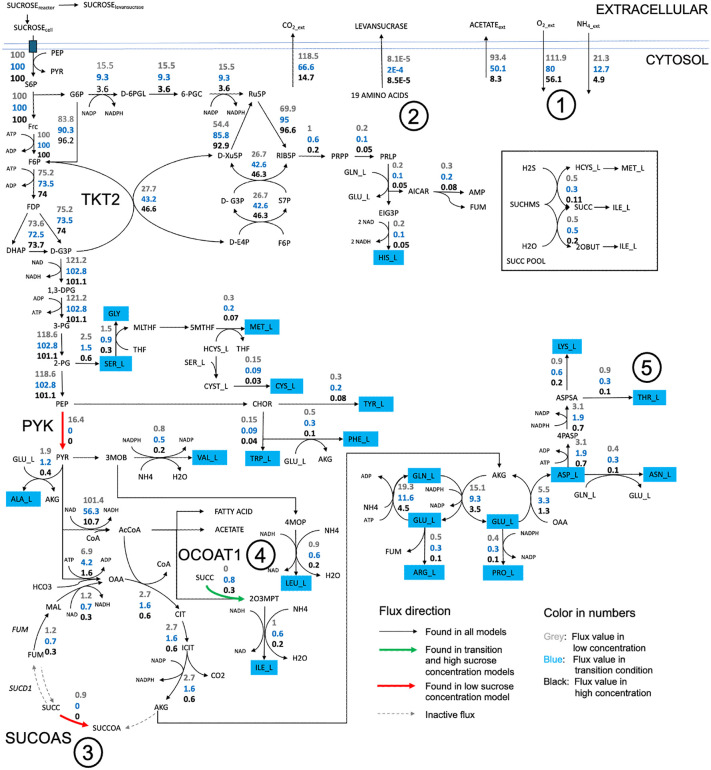
Simulation of metabolic flux conversion in *B. subtilis* LY7.16 under low (100 g·L^-1^), low-to-high transitional (200 g·L^-1^), and high (250 g·L^-1^) sucrose concentrations. The number values represent normalized fluxes expressed as flux-sums, enabling comparative analysis of metabolite fluxes across varying initial sucrose concentrations: low (grey), transitional low-to-high (blue), and high (black).

The metabolic model of *B. subtilis* LY7.16 was simulated under initial sucrose concentrations of 100 g·L ⁻ ¹ (low-range), 200 g·L ⁻ ¹ (transitional), and 250 g·L ⁻ ¹ (high-range). In each scenario, sucrose utilization was modulated based on the optimized biomass yield on substrate (*Y*_*X/S*_), ensuring alignment with the corresponding extracellular environment. Fig 6 presents the predicted metabolic flux distributions, illustrating metabolite conversion in *B. subtilis* LY7.16 across gradients of the initial sucrose concentrations. Fluxes are expressed as flux-sums (see Methods), facilitating comparative analysis across the different simulation conditions. The results indicate that microbial metabolism in *B. subtilis* LY7.16 prioritizes energy expenditure for levansucrase synthesis, while allocating fewer resources toward cellular biomass biosynthesis under higher sucrose concentrations. Simulations revealed a decline in extracellular oxygen levels (Fig 6, Number 1) with increasing initial sucrose concentrations. As oxygen plays a critical role in supporting aerobic bacterial growth, this reduction suggests a deceleration of metabolic activity relevant to biomass production under high-sucrose conditions. The simulated results correspond well with the observed decrease in maximum specific growth rate (*μ*_*max*_) under elevated sucrose levels.

Under high-sucrose conditions, *B. subtilis* LY7.16 exhibited a metabolic shift, diverting carbon flux away from biomass formation and toward levansucrase production to support enhanced levan biosynthesis (Fig 6, Number 2). Nevertheless, the simulation result of levansucrase secretion seemed higher in transition condition. This phenomenon may happen due to the rapid growth of biomass in transition condition compared to high sucrose concentration in the snapshot of FBA simulation (time = 9 h). Therefore, at that time, the secretion is higher on transition condition, but it does not represent the overall production of levan production, since the levan synthesis takes longer time in higher sucrose concentration. The altered succinate conversion from more energy intensive via succinyl CoA synthase (SUCOAS, red arrow, Number 3) under low sucrose concentration condition to energy-free reaction via 3-oxoacid CoA-transferase (OCOAT1, green arrow, Number 4) under high sucrose concentration condition supports the metabolic redirection corresponding to the sucrose-induced *sac*B gene expression.

### In silico analyses for enhancing levan production by B. subtilis LY7.16

Analysis of levan conversion across varying initial sucrose concentrations suggests that the optimal condition lies within the transitional range, specifically around 200 g·L ⁻ ¹. Simulations using the *ly716*-Bs-dMM model further revealed that levan production in *B. subtilis* LY7.16 can be enhanced under this condition by mitigating metabolic trade-offs, thereby promoting more efficient substrate utilization and maximizing product yield at a sustainable production rate. In this study, *in silico* analyses involving amino acid pathway interventions and systems-level metabolic engineering were conducted to provide predictive hypotheses that may help suggest strategic approaches for improving levan biosynthesis.

**Fig 7:**
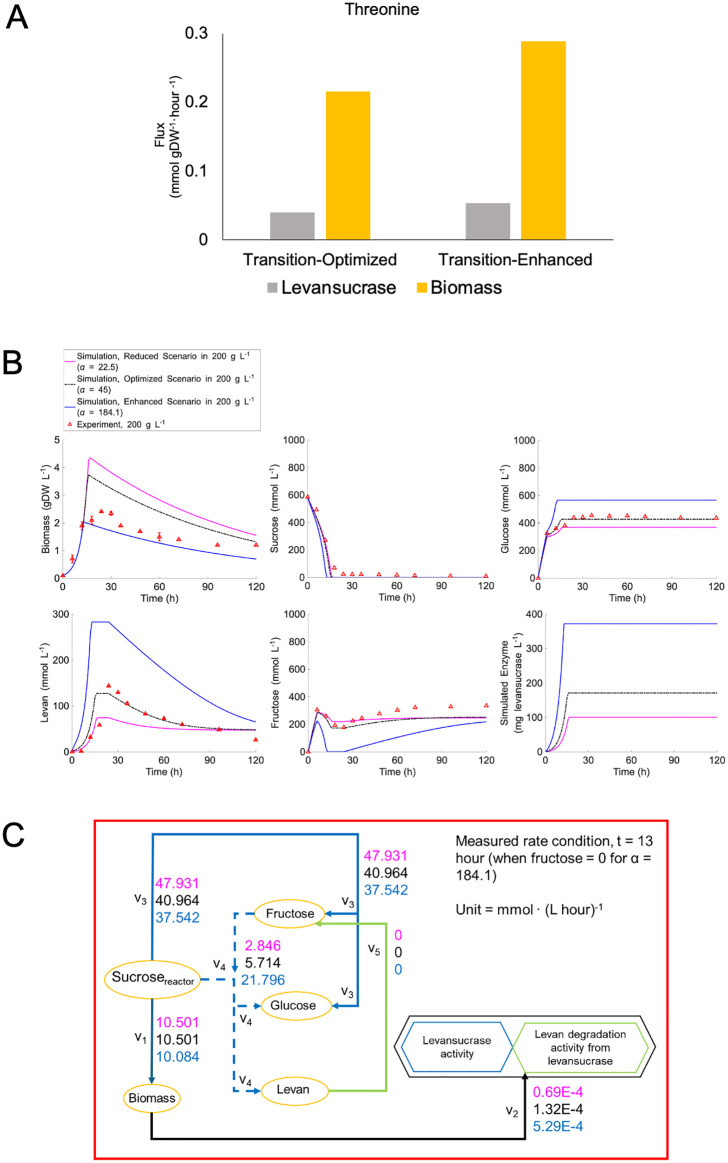
The levan enhancement scenario by *sacB* perturbation. (A) Simulation of levansucrase and cell biomass synthesis under threonine supplementation scenarios. (B) Simulation of the extracellular metabolite profiles, and (C) the corresponding conversion rate under altered levansucrase protein yield (*α* = 22.5 mg_levansucrase_ gDW_biomass_^-1^ – Pink, *α* = 45 mg_levansucrase_ gDW_biomass_^-1^ – Black, *α* = 184.1 mg_levansucrase_ gDW_biomass_^-1^ – Blue.

The first scenario was aimed to maximize levansucrase synthesis to evaluate the effect of amino acid supplementation on its production. Threonine was selected as a representative amino acid due to its distinct and differential production under low and transitional-to-high sucrose concentrations (Fig 6, Number 5). Through maximizing levansucrase synthesis, simulation in Fig 7A demonstrates a 1.342-fold increase in both levansucrase and biomass levels under this modification. These results suggest that targeted metabolic interventions, exemplified by threonine supplementation, may offer a promising strategy to redirect carbon flux toward a near-optimal state for microbial levansucrase production and levan synthesis.

An alternative strategy was explored based on the targeted metabolic engineering rationales. *In silico* perturbation of *sacB* was investigated through modulating levansucrase protein yield (*α* values) within the *ly716*-Bs-dMM framework and assess its impact on metabolic dynamics. Fig 7B shows simulations of levan production under reduced (*α* = 22.5 mg_levansucrase_·gDW_biomass_ ⁻ ¹), optimized (*α* = 45 mg_levansucrase_·gDW_biomass_ ⁻ ¹), and enhanced (*α* = 184.1 mg_levansucrase_·gDW_biomass_ ⁻ ¹) *α* values. A reduced *α* value led to increased biomass concentration but resulted in diminished levansucrase synthesis and lower levan output. Conversely, the enhanced *α* value stimulated levansucrase expression and levan production while suppressing biomass accumulation. Given that levan biosynthesis is constrained by substrate availability, *i.e.,* sucrose and fructose, these findings suggest that fed-batch cultivation may help maintain substrate supply, thereby enabling sustained levan production.

The simulation results presented in Fig 7B, along with the proposed strategy, are supported by the observed changes in sucrose consumption flux toward biomass production (*v*_*1*_), which decreases with increasing *α* values. This trend suggests a metabolic reallocation of carbon towards levan synthesis in *B. subtilis* at elevated *α* levels (Fig 7C). As a result, biomass formation is diminished under maximal α conditions, while levansucrase secretion and levan production are markedly enhanced, as shown in Fig 7B. Furthermore, the declining hydrolysis rate (*v*_*3*_) is associated with a reduced hydrolysis-to-transfructosylation (H/T) ratio, indicating a metabolic shift favoring transfructosylation activity in response to elevated levansucrase levels. Collectively, these results suggest that *sacB* overexpression, when combined with sustained substrate availability, may serve as an effective strategy to enhance and stabilize levan production.

**Fig 8:**
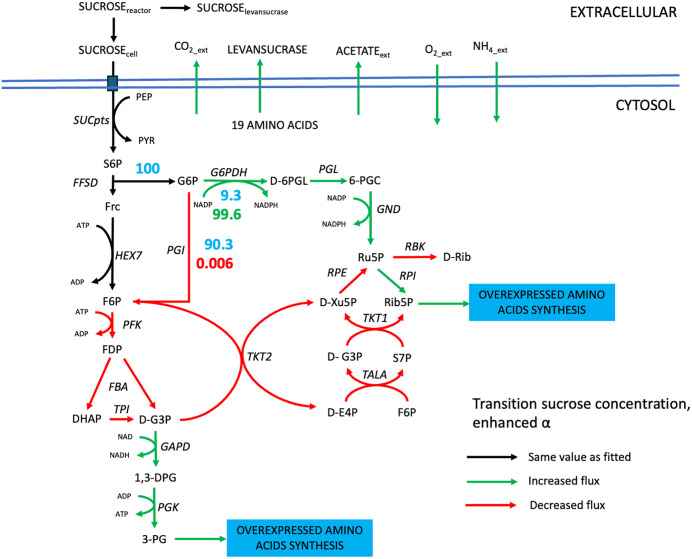
The proposed levan enhancement scenario using *ly716*-Bs-dMM framework by downregulating phosphoglucoisomerase *(PGI)* expression. Blue number indicating the original flux prior to *PGI* downregulation, while green number indicates an increasing shift towards *PGI* downregulation, and red number indicates the decreasing flux towards *PGI* knockdown.

Furthermore, the *ly716*-Bs-dMM framework demonstrated additional potential, as illustrated in Fig 8. Overexpression of *sacB* resulted in widespread enhancement of metabolic fluxes—indicated by the predominance of green pathways—while maintaining a growth rate comparable to the fitted value. However, Fig 8 highlights a flux reduction at the branching point between the oxidative pentose phosphate pathway (OPP) and glycolysis, implicating glucose-6-phosphate dehydrogenase (G6PDH) and phosphoglucoisomerase (PGI) as potential metabolic targets for further levansucrase enhancement. A simulation was performed in which PGI flux was downregulated to 0.006 mmol gDW_biomass_^-1^ h^-1^, successfully redirecting flux toward G6PDH and increasing NADPH generation, an energy exchange molecule. These findings suggested that a combined strategy involving *sacB* and G6PDH overexpression, PGI downregulation, and fed-batch cultivation may significantly enhance levan production.

## Discussion

Levan biosynthesis is governed by multiple factors, with initial sucrose concentration emerging as one of the most critical determinants. Elevated sucrose levels have been shown to enhance *sacB* gene expression and levansucrase secretion, thereby promoting levan production [5,13,16,35]. Kinetic studies based on Michaelis-Menten and Hill models have further corroborated the positive correlation between sucrose concentration and levan yield [36,37], providing a foundation for predictive modeling. The variation of levansucrase activity across sucrose concentrations was successfully modelled using a simple Heaviside step function, along with reparameterization of kinetic parameters expected to vary with sucrose concentration, such as *μ* and *α*. The model integrated existing knowledge of metabolite conversion kinetics to define plausible ranges for kinetic parameters, while also providing optimized estimates for reactions that remain less well characterized. The key kinetic parameters, including those derived from literature, were subsequently assessed through sensitivity analysis. The consistent simulation performance under 50% perturbations confirmed their plausibility and demonstrated that the model was not subject to overfitting. Among which, *α* seems to show high influence under high range of sucrose concentration. (Figs C-E in S1 Text).

Previous GEM-based studies on levan production in *Halomonas smyrnensis* AAD6T and *Bacillus subtilis* have pursued optimization through systems metabolic engineering [20,33]. These efforts, however, largely emphasized pathway-level interventions within GEM frameworks without accounting for dynamic metabolic changes over time—a critical consideration given that *B. subtilis* LY7.16 secretes extracellular levansucrase, which catalyzes levan synthesis outside the cell. To bridge this gap, we developed the *ly716*-Bs-dMM framework, which integrates dynamic modeling with GEM to simulate levansucrase kinetics and cellular metabolism. This approach extends the classical dFBA framework by incorporating both intra- and extracellular interconstraints. Rather than solely examining the dynamic shifts in intracellular metabolism in response to an evolving environment, the dynamic metabolic modeling of l*y716*-Bs-dMM framework conceptually emphasizes the reciprocal interplay between intracellular and extracellular processes.

The l*y716*-Bs-dMM framework successfully captured extracellular metabolite dynamics across varying sucrose concentrations (Figs 3–5). Notably, adjustments to the Michaelis–Menten formulation were required to incorporate fructose as a secondary substrate under high sucrose conditions, consistent with earlier reports [38,39]. The model also captured the dual role of levansucrase in both synthesis and degradation that often occurs particularly under sucrose-limited conditions [10,34]. While levan synthesis was slightly overestimated at low sucrose concentrations, glucose and fructose profiles were accurately reproduced, underscoring the robustness of the framework.

Intracellular flux analysis revealed a metabolic shift—particularly in succinate catabolism—indicating increased ATP demand for levansucrase synthesis (Fig 6). This observation aligns with prior findings on ATP competition between glycolysis and protein synthesis in *E. coli* [40]. The concomitant decline in growth rate alongside enhanced levan yield supports the hypothesis that energy is redirected from biomass formation toward levansucrase production, consistent with elevated *sacB* expression under high sucrose conditions [16,28]. Supplementation with amino acids, for example threonine, may mitigate biosynthetic burdens and sustain levansucrase synthesis without compromising growth.

Beyond metabolic insights, the *ly716*-Bs-dMM framework offers strategic value for systems metabolic engineering. By coupling dynamic modeling with GEM, it enables prediction of gene targets for enhancing levan biosynthesis. For example, *sacB* overexpression under transitional conditions (represented by *α* value modulation) revealed distinct flux redistribution patterns (Fig 8). The branching point at glucose-6-phosphate—directed either toward glycolysis via PGI or the oxidative pentose phosphate pathway (OPP) via G6PDH—was identified as a key regulatory node. Redirecting flux from *PGI* to *G6PDH*, as demonstrated in *Corynebacterium glutamicum* to enhance NADPH and L-arginine production [41], may similarly benefit levansucrase synthesis. These findings are supported by previous studies on NADPH-driven biosynthesis [42].

Our results also corroborate earlier modeling work suggesting that glycolysis-associated enzymes are critical for levan enhancement. While Immanuel et al. proposed knockout of *pfkA* and *pgk* [33], our simulations suggest that PGI knockdown effectively reduces flux to *pfkA*, whereas *pgk* flux behavior may vary due to its role in branching toward the non-oxidative pentose phosphate pathway. These discrepancies emphasize the necessity of integrating experimental validation with simulation-based predictions.

While the *ly716-*Bs-dMM framework demonstrates strong potential as a robust tool for enhancing levan production, its current application remains limited to the *B. subtilis* species level and has not yet fully achieved strain-specific representation for LY7.16. Moreover, the framework might provide more reliable predictions under nutrient-rich conditions. These constraints stem from the absence of a complete LY7.16 genome for model construction and the reliance on simulations optimized for nutrient broth (NB) with varying sucrose concentrations, which assume nutrient excess aside from sucrose variation. The present *ly716-*Bs-dMM framework was intentionally simplified to capture the dynamics of extracellular levan synthesis, leaving out several factors that may influence both the quality and quantity of levan. Looking forward, refinement of the framework could involve incorporating parameters related to levan product quality, such as molecular structural variations under different sucrose concentrations, as well as yield-enhancing downstream processes. For instance, ion-mediated precipitation (e.g., Mg²⁺ or Ca²⁺) is considered critical for both the quality and quantity of levan production and should be integrated into future model development.

In summary, the *ly716*-Bs-dMM framework offers a comprehensive platform for understanding and optimizing levan biosynthesis under diverse sucrose conditions. By capturing dynamic metabolic trade-offs and enabling targeted engineering strategies, it advances both mechanistic insight and practical application. Although current simulations are limited to batch culture, future extensions to fed-batch or continuous systems will be essential to improve industrial scalability and maximize yield alongside substrate use efficiency.

## Conclusions

This work highlights the importance of integrated framework to elucidate environmental effect and bacterial metabolism which are translated into the *ly716*-Bs-dMM. The *ly716*-Bs-dMM facilitated a comprehensive study of levan production performance and formulate the systems metabolic engineering strategies for levan production optimization. Simulation results revealed that the mechanism of levan production is optimally achieved with a high initial sucrose concentration combined with short time levan production. Systems metabolic engineering strategy, together with supplementary amino acids may work synergistically to boost levan production based on findings using the framework, *ly716-*Bs-dMM. The predictive hypotheses suggested the potential strategies that might be synergistically contributed to maximize levan yield with substrate efficiency that holds potential future applications with real-world industrial scenario.

## Materials and methods

### Materials

Fructose, glucose, sucrose, yeast extract, beef extract, and peptone were purchased from HiMedia Chemicals India Pvt. Ltd. (Mumbai, India), and all other reagents were obtained from Sigma-Aldrich (St. Louis, MO, USA). All chemicals were of analytical grade. Unless otherwise stated, all other reagents were obtained from Sigma-Aldrich (St. Louis, MO, USA).

### Microorganisms, media and culture conditions

*Bacillus subtilis* strain LY7.16 was isolated from Thai traditional fermented soybean and identified as previously described [18]. The 16S rRNA gene sequence was deposited in the GenBank database under accession number PV436786.1. The strain was deposited at the Thailand Bioresource Research Center (TBRC), Thailand, under accession number TBRC11053.

*B. subtilis* LY7.16 was maintained on nutrient agar (NA) containing peptone (5 g·L^-1^), NaCl (5 g·L^-1^), yeast extract (1.5 g·L^-1^), beef extract (1.5 g·L^-1^), and agar (20 g·L^-1^), and incubated aerobically at 37°C. For inoculum preparation, a loopful of culture was transferred into sterile nutrient broth (NB) with the same composition as NA without agar and incubated at 37°C with agitation at 200 rpm for 24 h. The culture was harvested when the optical density at 600 nm (OD₆₀₀) reached approximately 0.6, corresponding to a cell concentration of ~10⁸ colony-forming units (CFU)·mL^-1^, and used as the inoculum for subsequent fermentation experiments.

### Production of levan from sucrose by *B. subtilis* LY7.16

A 2% (v/v) inoculum of *B. subtilis* LY7.16 was transferred into nutrient broth (NB) medium supplemented with sucrose at concentrations of 50, 100, 200, 250, and 300 g·L^-1^. The medium was formulated to provide cells with sufficient nutrients from the complex culture environment, while ensuring that sucrose availability exerted a stronger influence on their growth and metabolism [2,5,13,14]. The cultures were incubated at 37°C with agitation at 200 rpm for 120 h. Samples were collected at 6, 12, 18, 24, 30, 36, 48, 60, 72, 96, and 120 h and centrifuged at 10,000 × g for 10 min to separate the cells from the culture supernatant. The concentrations of sucrose, glucose, and fructose in the culture supernatants were determined using high-performance liquid chromatography (HPLC) equipped with a refractive index detector (RID-10A; Shimadzu, Japan). Separation was performed on an Aminex HPX-42C column (Bio-Rad, Hercules, CA, USA) using HPLC-grade water (Fisher Scientific, UK) as the mobile phase at 85 °C and a flow rate of 0.6 mL·min^-1^.

For levan recovery, the cell-free supernatant was mixed with chilled 70% (v/v) ethanol at a ratio of 3:1 (ethanol to supernatant) and incubated at 4°C overnight. The precipitated levan was collected by centrifugation at 10,000 × g for 10 min. The resulting levan precipitate and the corresponding fresh cell pellets were dried to constant weight in an oven at 50°C to determine levan yield and dry cell biomass, respectively.

### Construction of *ly716*-Bs-dMM model

A dynamic metabolic model of *B. subtilis* LY7.16 (*ly716*-Bs-dMM) was developed by integrating an ordinary differential equation (ODE)-based kinetic model (denoted as BsODE) with a constraint-based genome-scale metabolic model (denoted as BsCBM). This hybrid approach was employed to simulate microbial growth and dynamic changes of metabolites over time under different initial sucrose concentrations. The model combined kinetics of extracellular biochemical reactions with genome-scale metabolic flux distributions inside microbial cells to investigate conversion of sucrose into levan under different initial substrate concentrations. In the model, sucrose consumption was assumed to follow Michaelis–Menten, and growth of microbial cell was assumed to exhibit Monod kinetics [34,36,43].

The BsODE-based kinetic model was developed to describe the time-dependent dynamics of biomass growth over time, which is represented as $\frac{dX}{dt}$, sucrose consumption, which is represented as *v*_*1*_, and metabolite production in batch culture. It was constructed based on prior knowledge of levansucrase synthesis and relevant literature. Experimental data demonstrated that initial sucrose concentration markedly affected levan yield and the synthesis of glucose and fructose (Fig 1). These trends were related to levansucrase enzymatic activities — hydrolysis, transfructosylation, and levan degradation — represented as *v*_*3*_*-v*_*5*_ in Fig 2A. The hydrolysis/transfructosylation (H/T) ratio has been reported to inversely correlate with sucrose concentration [22,26].

Accordingly, BsODE was designed to simulate biomass growth, sucrose uptake, and product formation (levan, fructose, and glucose) based on the dynamic mass balance (Fig 2B). Levansucrase, which is an extracellular enzyme, catalyzed levan synthesis; thus, levan formation was assumed to occur extracellularly in the presence of sucrose and levansucrase, consistent with prior findings [2,6,44]. The system dynamics are described by the ODE model in Equations (1–7), with the related kinetic functions detailed in Equations (8–11).


$$\begin{array}{c} \frac{dX}{dt}=\left(\mu -k_{d}\right)X\; \end{array}$$ (1)

$$\begin{array}{c} \frac{{dSucrose}_{cell}}{dt}{=-v}_{\text{1}}=-\frac{\mu X}{Y_{X/S}}\; \end{array}$$ (2)

$$\begin{array}{c} \frac{dLevansucrase}{dt}=\;v_{\text{2}}=\alpha *\mu *X\; \end{array}$$ (3)

$$\begin{array}{c} \frac{dGlucose}{dt}=v_{\text{3}}+\left(v_{\text{4}}*\alpha *X\right)\; \end{array}$$ (4)

$$\begin{array}{c} \frac{dFructose}{dt}=v_{\text{3}}+\left(-v_{\text{4}}+\;\left(v_{\text{5}}*heaviside\left(t-t_{levan\;degradation}\right)\right)\right)(\alpha *X)\; \end{array}$$ (5)

$$\begin{array}{c} \frac{dLevan}{dt}=\left(v_{\text{4}}-\left(v_{\text{5}}*heaviside\left(t-t_{levan\;degradation}\right)\right)\right)(\alpha *X) \end{array}$$ (6)

$$\begin{array}{c} \frac{d{Sucrose}_{reactor}}{dt}=\;-v_{\text{1}}-v_{\text{3}}-\left(v_{\text{4}}*\alpha *X\right)\; \end{array}$$ (7)

$$\begin{array}{c} \mu =\frac{\mu_{max}\cdot [{Sucrose}_{reactor}]}{K_{s}+[{Sucrose}_{reactor}]} \end{array}$$ (8)

$$\begin{array}{c} v_{\text{3}}=\frac{v_{max,hyd,levansucrase}\;*\;{[Sucrose}_{reactor}]}{K_{M,hyd,levansucrase}\;+\;{[Sucrose}_{reactor}]}\; \end{array}$$ (9)

$$\begin{array}{c} v_{\text{4}}=\frac{v_{max,trans,levansucrase\text{1}\;}\frac{\left[{Sucrose}_{reactor}\right]}{K_{M,trans,levansucrase\text{1}}}\;+\;v_{max,trans,levansucrase\text{2}\;}\frac{\left[Fructose]\;*\;[{Sucrose}_{reactor}\right]}{K_{M,trans,levansucrase\text{2}}}}{\text{1}+\;\frac{\left[{Sucrose}_{reactor}\right]}{K_{M,trans,levansucrase\text{1}}}\;+\;\frac{\left[Fructose]\;*\;[{Sucrose}_{reactor}\right]}{K_{M,trans,levansucrase\text{2}}}}\; \end{array}$$ (10)

$$\begin{array}{c} v_{\text{5}}=\frac{v_{max,levan\;degradation}\;*\;\left[Levan-(\text{0}.\text{33 }*\;Levan)\right]}{K_{M,levan\;degradation}+\left[Levan-(\text{0}.\text{33 }*\;Levan)\right]}\; \end{array}$$ (11)

*µ*is specific growth rate (h^-1^), *µ*_*max*_ is maximum specific growth rate (h^-1^), and *k*_*d*_ is death constant (h^-1^). The levansucrase-associated reaction, *v*_*3*_-*v*_*5*_ also includes the enzymatic parameters *v*_*max,hyd,levansucrase*_ (mmol·mg_levansucrase_^-1^·h^-1^), representing maximum hydrolysis rate; *v*_*max,trans,levansucrase*_ (mmol·mg_levansucrase_^-1^·h^-1^) representing maximum transfructosylation rate; and *v*_*max,levan degradation*_ (mmol·mg_levansucrase_^-1^·h^-1^), representing maximum levan degradation rate. Each reaction is further characterized by its Michaelis–Menten constant (*K*_*M, hyd, levansucrase*_*, K*_*M, trans, levansucrase*_*, K*_*M, levan degradation*_) expressed in mM.

Auxiliary parameters were incorporated to describe the metabolic shifts under different sucrose levels. These included the biomass yield on sucrose (*Y*_*x/s*_) and *α*, representing the levansucrase production yield (mg) on basis of a gram dry weight of biomass (gDW_biomass_). A Heaviside function and reparameterization of kinetic parameters were employed to introduce sucrose-dependent activity of levansucrase across low-to-high sucrose concentrations regime [45], particularly simulating degradation of levan in an absence of sucrose. The onset of levan degradation (*t*_*levan degradation*_) was used as a parameter constraining levan synthesis activity. The parameter *α* was embedded in the biomass growth function to estimate levansucrase secretion, as levansucrase was considered a growth-associated metabolite [ 46].

The BsCBM model was reconstructed from the genome-scale metabolic model *i*YO844 of *B. subtilis* 168, as shown evolutionary closed to *B. subtilis* LY7.16 based on high 16S rRNA gene sequence similarity (S3 Text). Levansucrase-related reactions absent in *i*YO844 were incorporated to reflect *B. subtilis* LY7.16’s capacity for levan polymerization [8,47,48]. The BsCBM model was further refined to ensure mass balance and network completeness, with manual gap-filling based on KEGG and MetaCyc and literature evidence [47,49]. The model curation and analysis were performed using COBRA Toolbox v3.0 [50] implemented in MATLAB (R2023b, The MathWorks). The BsCBM framework was calibrated to simulate the growth of *Bacillus subtilis* LY7.16 under varying sucrose concentrations in levan production experiments, ensuring that the model captured the metabolic behavior of this strain. This strategy enabled simulation of metabolic conversion in metabolism of *B. subtilis LY7.16* despite the absence of genome sequence data, though potential strain-specific responses may not be fully represented. Each simulation run using the *ly716*-Bs-dMM framework took approximately 10 minutes on a laptop computer equipped with an AMD Ryzen 4700U processor and 8 GB of memory.

The two submodels, BsODE and BsCBM, were coupled via the constraining linker parameters from BsODE (*μMonod*, *Y*_*X/S*_*,* and *α*) to BsCBM, resulting in the integrated *ly716*-Bs-dMM model. The details of the submodel integration are illustrated in Fig 2B. The integrated framework, *ly716-*Bs-dMM, along with the condition-specific data and the script for running *ly716-*Bs-dMM, is available in the GitHub repository KMUTT-CASB/ ly716-Bs-dMM (https://github.com/KMUTT-CASB/ly716-Bs-dMM).

### Model simulation

To investigate the dynamic behavior of *B. subtilis* LY7.16, simulations were performed at initial sucrose concentrations of 100, 200, and 250 g·L^-1^, representing low, transition, and high levels, respectively. Parameter estimation within BsODE was achieved by minimizing the sum of squared errors between model predictions and experimental data using the Levenberg–Marquardt algorithm [51]. All kinetic parameters were independently estimated for the low and high sucrose conditions, except for levansucrase activity parameters, which were held constant. The transition condition (200 g·L^-1^) was simulated using interpolated parameters between the two conditions to assess intermediate systems dynamics. Estimated parameters and their fitted values are summarized in the S2 Text.

Intracellular flux distributions were simulated using flux balance analysis (FBA), a constraint-based optimization technique estimating steady-state metabolic fluxes from stoichiometric and physiological constraints. In the *ly716*-Bs-dMM model, BsCBM was constrained using auxiliary parameters derived from BsODE, including *μMonod* (h^-1^), sucrose uptake rate (mmol gDW_biomass_^-1^ · h^-1^), and specific levansucrase yield (*α*, mg_levansucrase_⋅gDW_biomass_^-1^). Model was optimized towards minimal total flux [ 52,53] to examine metabolic flux distributions across sucrose variations, ensuring the representation of suboptimal growth states where nutrient utilization was partitioned between biomass formation and levansucrase secretion [30,32,33]. Simulation focused on mid-exponential growth phase (9 h). The complete mathematical formulation is given in Equations 12–16.


$$\begin{array}{c} \mu_{Monod}=\;\frac{\text{1}}{X}\;\left(\frac{dX}{dt}\right) \end{array}$$ (12)

$$\begin{array}{c} s.\;t.\;{\;\mu }_{BsCBM}=\;\mu_{Monod} \end{array}$$ (13)

$$\begin{array}{c} s.\;t.\;{\;flux}_{sucrose\;uptake,\;BsCBM}=\;{flux}_{sucrose\;uptake,\;BsODE} \end{array}$$ (14)

$$\begin{array}{c} s.\;t.\;{\;\alpha }_{BsCBM}=\;\alpha_{BsODE},\;s.\;t. \end{array}$$ (15)

$$\begin{array}{c} \left[\begin{array}{c} \;\begin{array}{c} \text{min}\sum_{j=\text{1}}^{m}\;\left|{flux}_{\;j}\right|\\ f\\ s.t.\;S_{i,j}\cdot \;{flux}_{j}=\text{0} \end{array}\\ f\\ \;\text{0}\leq \;{flux}_{j}\;\leq \;{flux}_{j,max\;}\; \end{array}\right]\; \end{array}$$ (16)

The stoichiometric matrix *S*_*irrev,i,j*_ contains the stoichiometric coefficient of metabolite *i* in reaction *j.* Subscription “*m*” represents total metabolite, while “*i”* represents a specific metabolite. The variable *flux*_*irrev,j,*_ represents intracellular fluxes within the metabolic model, ranging from 0 to a pre-defined upper bound (*flux*_*j,max*_). The subscript “*irrev”* indicates that all reactions were converted to irreversible form to minimize the total absolute flux [54].

### Model validation

The model was validated against measured data from independent experiments. The structure and associated parameters of the *ly716*-Bs-dMM model were evaluated using experiments with initial sucrose concentrations of 50 g·L⁻^1^ and 300 g·L⁻^1^, representing the low and high modeling conditions, respectively. Normalized root mean squared error (NRMSE) was used to quantify the deviation between predicted and observed metabolite profiles, thereby assessing the predictive reliability of the model.

### Model analysis

Flux distribution analyses were performed to assess metabolic adaptations under different sucrose levels. Flux-sum analysis was used to normalize each metabolite’s flux by the sucrose uptake rate [55]. Reactions with flux values less than 1 × 10⁻^6^ mmol gDW_biomass_⁻^1^ h⁻^1^ were considered inactive [56]. Fluxes were categorized as (i) active across the whole range of sucrose concentrations, (ii) active only at low range of sucrose concentrations, (iii) active at transition and high range of sucrose concentrations, or (iv) inactive under all conditions. Differences in active fluxes between conditions were analyzed to understand mechanisms underlying variable levan synthesis performance.

### In silico analysis of levan production enhancement

The ly716-Bs-dMM framework was used to explore strategies for improving levan yield at a transitional sucrose concentration (200 g·L^-1^). Three simulation scenarios were conducted: (i) maximizing levansucrase synthesis by modified the objective function to assess amino acid concentrations to levansucrase production; (ii) examin*ing* the effect of sacB downregulation and overexpression on extracellular metabolite patterns *by* varying α to; and (iii) investigating the NADPH-rich metabolic pathways to evaluate intracellular cofactor balance impacted on levan biosynthesis. These analyses provided a comprehensive system-level scenario of environmental and intracellular factors influencing levan production.

## Supporting information

S1 Text Levan production, metabolite profile experimental data and global sensitivity analysis. (DOCX)

S2 Text List of model parameters and condition-specific optimized values. (DOCX)

S3 Text BLASTn pairwise comparative genome analysis of *Bacillus subtilis* LY7.16 using 16S rRNA. (DOCX)
