# Supplementary material for: Dynamic metabolic modeling uncovers systems-level strategies to simultaneously maximize levan yield and substrate efficiency in Bacillus subtilis LY7.16
Source: PLoS Comput Biol. 2026 May 18;22(5):e1014273. doi: 10.1371/journal.pcbi.1014273 (PMC13197064; doi:10.1371/journal.pcbi.1014273)
Supplement: S1 Text — (DOCX) [file pcbi.1014273.s001.docx]

**S1 Text. Levan production, metabolite profile experimental data and global sensitivity analysis**

In the development of the *ly716*-Bs-dMM model, experimental data of levan production and metabolite profiles in batch culture system were performed by the Excellent Center of Enzyme Technology and Microbial Utilization, Pilot Plant Development and Training Institute, King Mongkut’s University of Technology Thonburi, Thailand. Figure S1 presents the experimental measured data, including biomass accumulation, sucrose consumption, and product formation (glucose, levan, and fructose). Figure S2 shows the performance of the *ly716*-Bs-dMM framework when applied to a higher sucrose concentration (250 g·L^-1^). Since there were numerous kinetic parameters that being applied in *ly716-*Bs-dMM, the global sensitivity analysis was carried out to observe the sensibility of parameters within the model, particularly for parameter which were obtained from literature without any adjustment by model fitting (Supplementary Table S2). Figure S3-S5 showed the global sensitivity analysis when *K_m_*  for *v_5_*, μ_max_ and α values are increased and decreased by 50%, respectively, which were being predicted using 50 and 300 g L^-1^ of initial sucrose concentration.

**Figure A** Time-course measurement of metabolite profiles from levan production via *Bacillus subtilis* LY7.16 under batch conditions. The measurements include A) biomass concentration, B) sucrose consumption, C) glucose formation, D) levan production, and E) fructose formation at different initial sucrose concentrations (50, 100, 200, 250, and 300 g L^-1^). Data were performed by the Excellent Center of Enzyme Technology and Microbial Utilization, Pilot Plant Development and Training Institute, King Mongkut’s University of Technology Thonburi, Thailand.


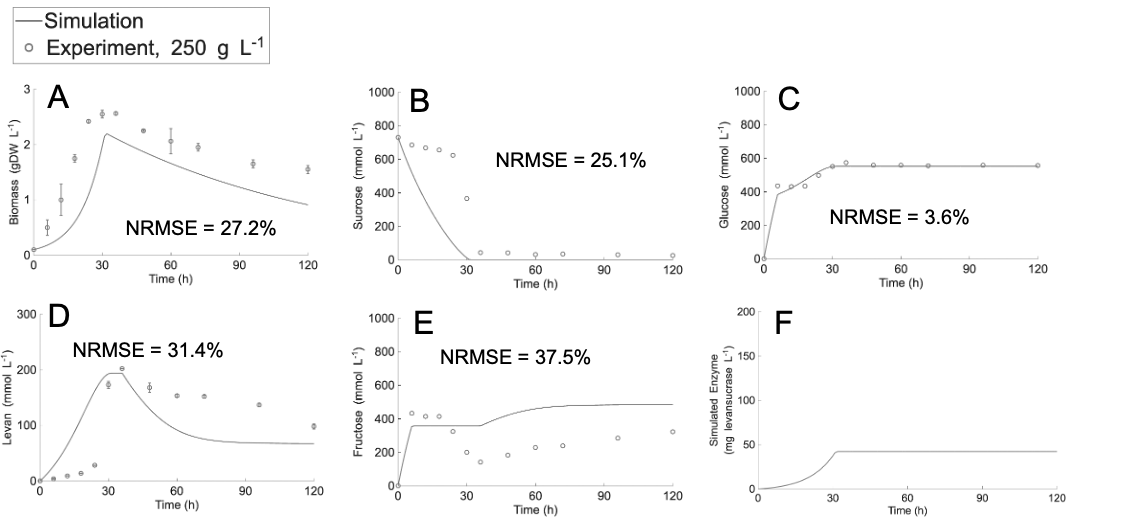


**Figure B** Simulation of levan production and metabolite profiles under high sucrose concentration (250 g·L⁻¹) using *ly716*-Bs-dMM framework constructed based upon the low-sucrose conditions (100 g·L⁻¹). The simulation includes A) biomass concentration, B) sucrose consumption, C) glucose formation, D) levan production, E) fructose formation and F) simulated levansucrase concentration


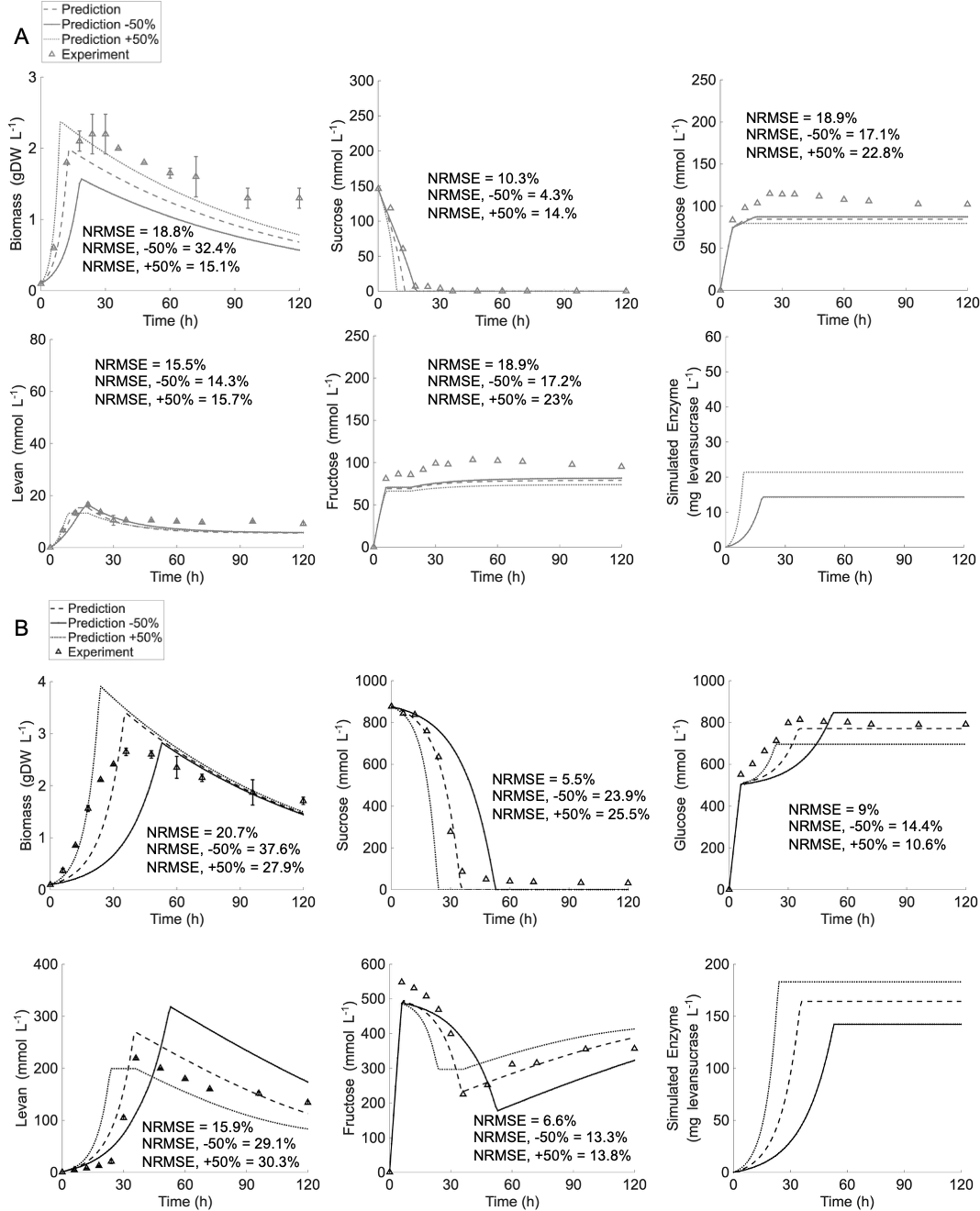


**Figure C** Prediction of levan production and metabolite profiles under high sucrose concentration (300 g·L⁻¹) using *ly716*-Bs-dMM framework constructed based upon the low-sucrose conditions (50 g·L⁻¹). The global sensitivity analysis was carried out to observe the sensibility of measured μ_max_ with the range of 50% increasing and decreasing from original value, respectively.


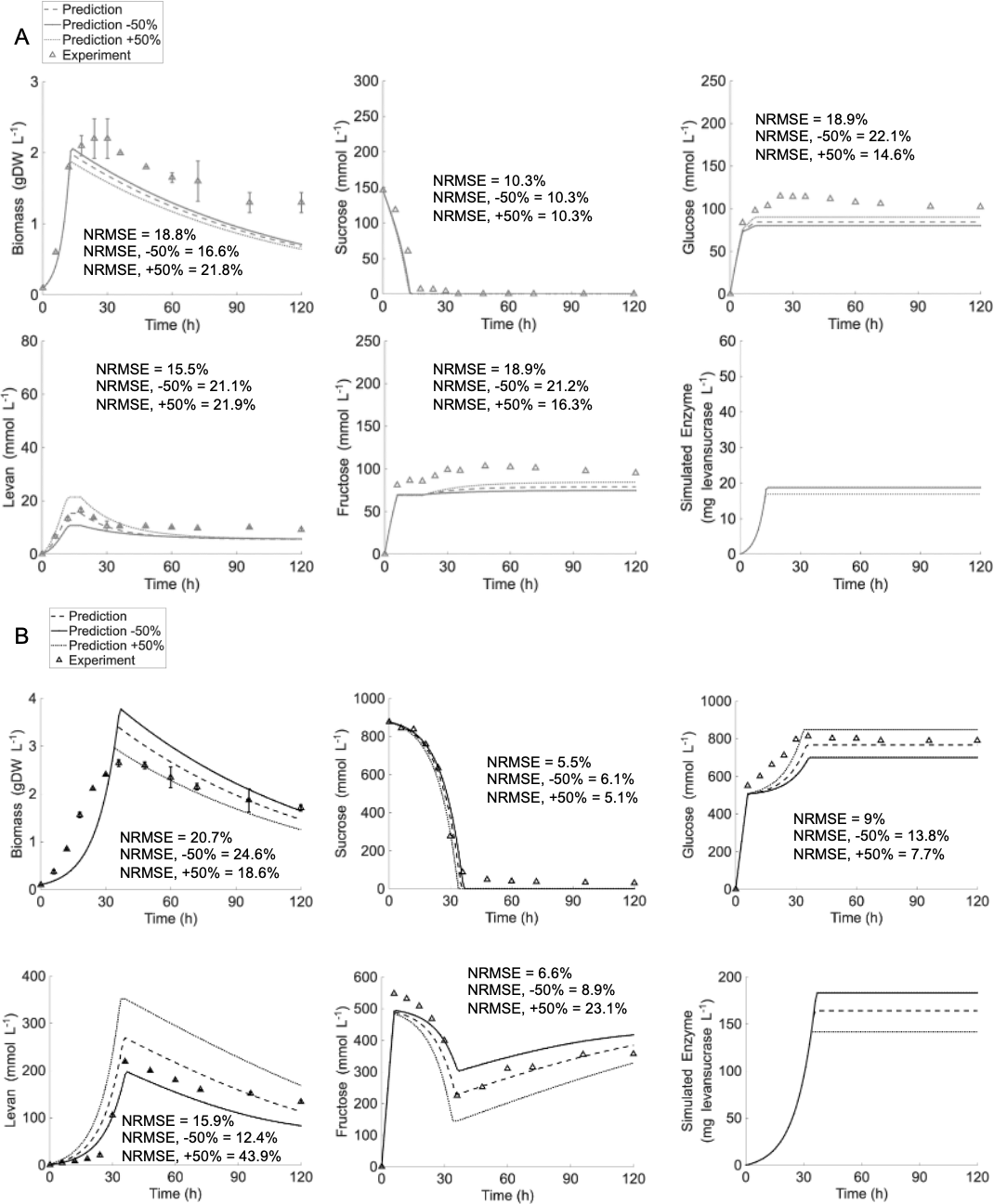


**Figure D** Prediction of levan production and metabolite profiles under high sucrose concentration (300 g·L⁻¹) using *ly716*-Bs-dMM framework constructed based upon the low-sucrose conditions (50 g·L⁻¹). The global sensitivity analysis was carried out to observe the sensibility of fitted α values with the range of 50% increasing and decreasing from original value, respectively.


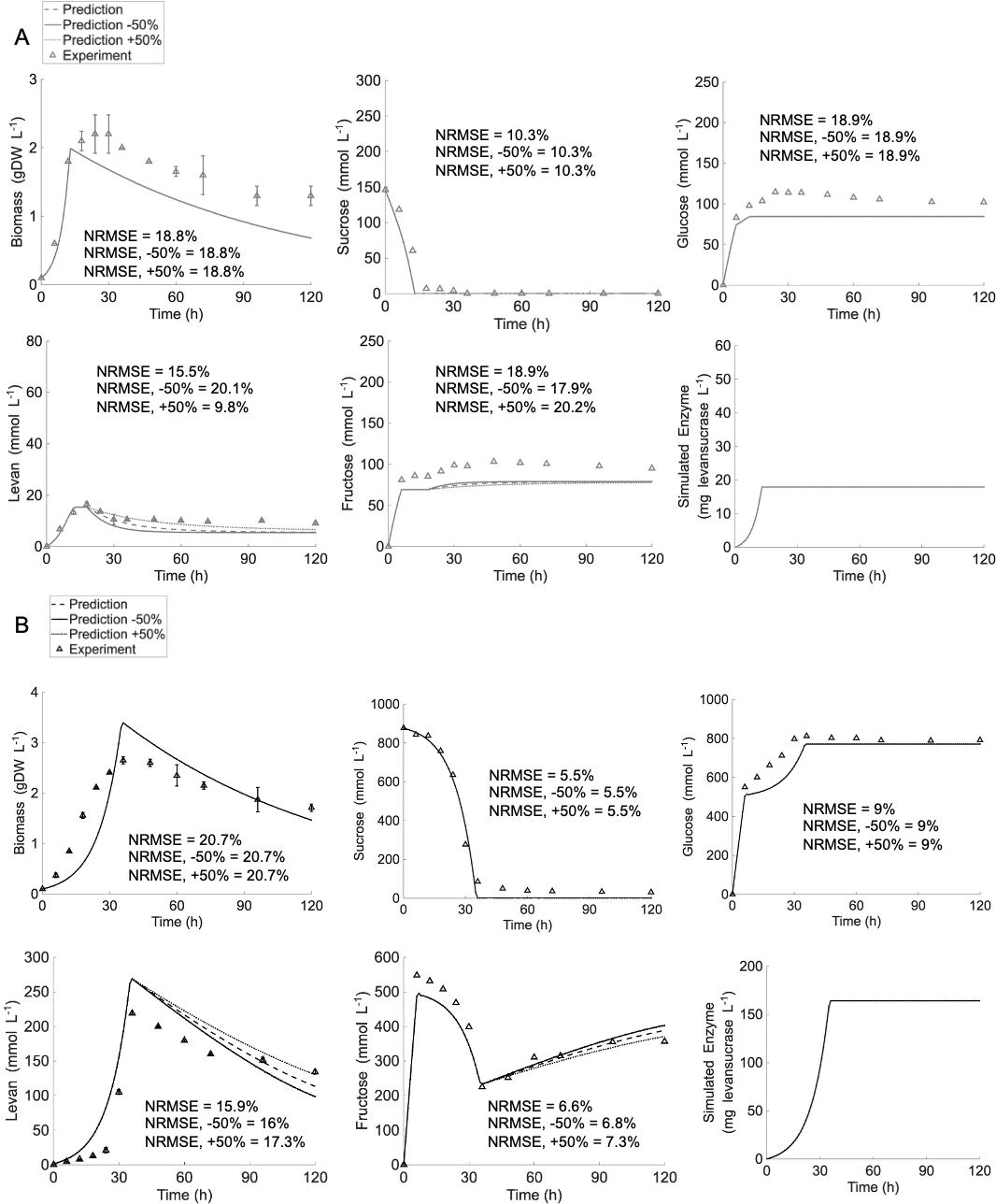


**Figure E** Prediction of levan production and metabolite profiles under high sucrose concentration (300 g·L⁻¹) using *ly716*-Bs-dMM framework constructed based upon the low-sucrose conditions (50 g·L⁻¹). As *Km* for *v5* was obtained from literature, global sensitivity analysis was carried out to observe the sensibility of obtained parameter with the range of 50% increasing and decreasing from original value, respectively.
