# Supplementary material for: Dynamic metabolic modeling uncovers systems-level strategies to simultaneously maximize levan yield and substrate efficiency in Bacillus subtilis LY7.16
Source: PLoS Comput Biol. 2026 May 18;22(5):e1014273. doi: 10.1371/journal.pcbi.1014273 (PMC13197064; doi:10.1371/journal.pcbi.1014273)
Supplement: S2 Text — (DOCX) [file pcbi.1014273.s002.docx]

**S2 Text: List of model parameters and condition-specific optimized values**

The BsODE model parameters and their optimized values at particular conditions are summarized in Table S1. These parameters were employed to represent the characteristics of each mechanistic occurrence of levan production under varying range of sucrose concentration.

Table A Parameters and corresponding values used in the BsODE model of Bacillus subtilis LY7.16 under different initial sucrose concentrations.

| Parameters | Description | Parameter optimized by  ly716-Bs-dMM model | | | Parameter ranges reported in literature | Unit | Source of parameters | References |
| --- | --- | --- | --- | --- | --- | --- | --- | --- |
|  |  | Low sucrose | Transition sucrose | High sucrose |  |  |  |  |
| $\boldsymbol{\mu}_{\boldsymbol{max}}$ | Maximum specific of growth rate | 0.2454 | 0.2454 | 0.11 | - | h^-1^ | Measured |  |
| $\boldsymbol{k}_{\boldsymbol{d}}$ | Death constant | 0.01 | 0.01 | 0.01 | - | h^-1^ | Measured |  |
| α | Ratio of levansucrase produced per gDW of biomass | 9.095 (50 g L^-1^); 18.19 (100 g L^-1^) | 45 (200g L^-1^) | 45 (250 and  300 g L^-1^) | - | mg levansucrase·  gDW^-1^ | Optimized |  |
| $\boldsymbol{k}_{\boldsymbol{s}}$ | Monod constant | 0.75 | 0.75 | 0.75 | - | mM | Optimized |  |
| $\boldsymbol{v}_{\boldsymbol{max}}\boldsymbol{for}\boldsymbol{v}_{\boldsymbol{3}}$ | Maximum rate of levansucrase for hydrolysis reaction | 0.70 | 0.70 | 0.70 | 0.648-40.176* | mmol·  mg_levansucrase_ ^-1^ ·h^-1^ | Optimized | [1, 2] |
| $\boldsymbol{k}_{\boldsymbol{M}}\boldsymbol{for}\boldsymbol{v}_{\boldsymbol{3}}$ | Michaelis-Menten constant of levansucrase for hydrolysis reaction | 7.30 | 7.30 | 7.30 | 7.3-820.75 | mM | Optimized | [1, 2] |
| $\boldsymbol{v}_{\boldsymbol{max}}\boldsymbol{for}\boldsymbol{v}_{\boldsymbol{4}}$**,**  *sucrose* **(**denoted as *v_max,trans,levansucrase1_***_)_** | Maximum rate of levansucrase for transfructosylation reaction | 6.42 | 6.42 | 6.42 | 0.648-40.176* | mmol·  mg_levansucrase_ ^-1^ ·h^-1^ | Optimized | [1, 2] |
| $\boldsymbol{k}_{\boldsymbol{M}}\boldsymbol{for}\boldsymbol{v}_{\boldsymbol{4}}\boldsymbol{,}$  Sucrose (denoted as k_M,trans,levansucrase1)_ | Michaelis-Menten constant of levansucrase for transfructosylation reaction | 1,546.45 | 1,546.45 | 1,546.45 | 113.13-1,556.4 | mM | Optimized | [1, 2] |
| $\boldsymbol{v}_{\boldsymbol{max}}\boldsymbol{for}\boldsymbol{v}_{\boldsymbol{4}}$**,**  Fructose (denoted as v_max,trans,levansucrase2)_ | Maximum rate of levansucrase for transfructosylation reaction | - | 0.167 | 0.167 | 0.648-40.176* | mmol·  mg_levansucrase_ ^-1^ ·h^-1^ | Optimized | [1, 2] |
| $\boldsymbol{k}_{\boldsymbol{M}}\boldsymbol{for}\boldsymbol{v}_{\boldsymbol{4}}\boldsymbol{,}$  Fructose (denoted as k_M,trans,levansucrase2)_ | Michaelis-Menten constant of levansucrase for transfructosylation reaction | - | 1,533.6 | 1,533.6 | 113.13-1,556.4 | mM | Optimized | [1, 2] |
| $\boldsymbol{v}_{\boldsymbol{max}}\boldsymbol{for}\boldsymbol{v}_{\boldsymbol{5}}$ | Maximum rate of levansucrase for levan degradation reaction | 0.25 | 0.25 | 0.25 | 0.018-59.22* | mmol·  mg_levansucrase_ ^-1^ ·h^-1^ | Optimized | [2, 3] |
| $\boldsymbol{k}_{\boldsymbol{M}}\boldsymbol{for}\boldsymbol{v}_{\boldsymbol{5}}$ | Michaelis-Menten constant of levansucrase for levan degradation reaction | 63 | 63 | 63 | 0.0012-63.4 | mM | Estimated | [2, 3] |
| $\boldsymbol{Y}_{\frac{\boldsymbol{X}}{\boldsymbol{S}}}$ | Yield biomass per sucrose consumed | 0.025 | 0.015 | 0.006 | - | gDW biomass·  mmol _sucrose_ ^-1^ | Optimized |  |

*v_max_ is multiplied as 0.06 as factor conversion from μmol (min^-1^ mg^-1^) to mmol (hour^-1^ mg^-1^).

** Measured = Experimental measurement in this work, Optimized = Optimized by ly716-Bs-dMM model, and Estimated = Estimated by literature.

**References**

1. Tian, F., Inthanavong, L., & Karboune, S. (2011). Purification and characterization of levansucrases from Bacillus amyloliquefaciens in intra-and extracellular forms useful for the synthesis of levan and fructooligosaccharides. *Bioscience, biotechnology, and biochemistry*, *75*(10), 1929-1938.
2. Brenda-enzymes.org. <https://www.brenda-enzymes.org/>. Accessed on February 21, 2026.
3. Méndez-Lorenzo, L., Porras-Domínguez, J. R., Raga-Carbajal, E., Olvera, C., Rodríguez-Alegría, M. E., Carrillo-Nava, E., ... & López Munguía, A. (2015). Intrinsic levanase activity of Bacillus subtilis 168 levansucrase (SacB). *PloS one*, *10*(11), e0143394.
