# Supplementary material for: Dynamic metabolic modeling uncovers systems-level strategies to simultaneously maximize levan yield and substrate efficiency in Bacillus subtilis LY7.16
Source: PLoS Comput Biol. 2026 May 18;22(5):e1014273. doi: 10.1371/journal.pcbi.1014273 (PMC13197064; doi:10.1371/journal.pcbi.1014273)
Supplement: S3 Text — (DOCX) [file pcbi.1014273.s003.docx]

**S3 Text. BLASTn pairwise comparative genome analysis of *Bacillus subtilis* LY7.16 using 16S rRNA**

Pairwise alignment of the 16S rRNA gene sequence from *Bacillus subtilis* LY7.16 was performed against *Bacillus* species available in the NCBI 16S rRNA database for Bacteria and Archaea using the BLASTn [1]. The criteria and parameters used for the BLASTn analysis are summarized in Table S1.

Table A Parameters and criteria for BLASTn analysis of the 16S rRNA sequence of B. subtilis LY7.16.

| **Parameter** | **Criteria or setting** |
| --- | --- |
| Database | 16S ribosomal RNA sequences (Bacteria and Archaea) |
| Program selection to be optimized | Highly similar sequences (megablast) |
| Maximum target sequences | 10 |
| Expect threshold (E-value) | 0.05 (default) |
| Match/mismatch scores | 1 / -2 (default) |
| Gap costs | Linear (default) |

The BLASTn results indicate that the 16S rRNA sequence of *B. subtilis* LY7.16 exhibited a high degree of similarity with several *Bacillus* species. The query cover values indicated that the full-length 16S rRNA sequence of *B. subtilis* LY7.16 was completely aligned with all compared sequences. Furthermore, all alignments displayed an E-value of zero, confirming that the observed similarities were not due to random chance (Table S2). Although minor variations in percent identity were observed among species, all values exceeded 99%, which is well above the threshold of 98.7-99.0% recommended for species-level similarity in 16S rRNA sequence analysis [2]. Considering both the high sequence identity and the availability of a well-curated genome-scale metabolic model (GEM), *B. subtilis* 168 was selected as the reference strain for constructing the *B. subtilis* LY7.16 GEM (denoted as BsCBM).

Table B Top ten BLASTn pairwise comparison results for the 16S rRNA sequence of B. subtilis LY7.16.

| **Description** | **Sequence type** | **Query coverage**  **(%)** | **E-value** | **Percent identity (%)** |
| --- | --- | --- | --- | --- |
| *B. subtilis* strain DSM 10 | partial sequence | 100 | 0 | 100 |
| *B. subtilis* strain JCM 1465 | partial sequence | 100 | 0 | 100 |
| *B. subtilis* strain NBRC 13719 | partial sequence | 100 | 0 | 100 |
| *B. inaquosorum* strain BGSC 3A28 | partial sequence | 100 | 0 | 99.93 |
| *B. subtilis* strain IAM 12118 | complete sequence | 100 | 0 | 99.93 |
| *B. tequilensis* strain 10b | partial sequence | 100 | 0 | 99.93 |
| *B. subtilis* strain BCRC 10255 | partial sequence | 100 | 0 | 99.93 |
| *B. subtilis subsp. subtilis* strain 168 | complete sequence | 100 | 0 | 99.85 |
| *B. spizizenii* strain NRRL B-23049 | partial sequence | 100 | 0 | 99.85 |
| *B. spizizenii* strain NBRC 101239 | partial sequence | 100 | 0 | 99.85 |

**References**

1. Huser, B. A., Wuhrmann, K., & Zehnder, A. J. (1982). Methanothrix soehngenii gen. nov. sp. nov., a new acetotrophic non-hydrogen-oxidizing methane bacterium. *Archives of Microbiology*, *132*, 1-9.
2. Schlaberg, R., Simmon, K. E., & Fisher, M. A. (2012). A systematic approach for discovering novel, clinically relevant bacteria. *Emerging infectious diseases*, *18*(3), 422.
